# Enabling High Throughput Kinetic Experimentation by Using Flow as a Differential Kinetic Technique**

**DOI:** 10.1002/anie.202318146

**Published:** 2023-12-22

**Authors:** Gavin Lennon, Paul Dingwall

**Affiliations:** ^1^ School of Chemistry and Chemical Engineering Queen's University Belfast David Keir Building, Stranmillis Road Belfast BT9 5AG UK

**Keywords:** Aldol Reaction, Flow Chemistry, Kinetics, Organocatalysis

## Abstract

Kinetic data is most commonly collected through the generation of time‐series data under either batch or flow conditions. Existing methods to generate kinetic data in flow collect integral data (concentration over time) only. Here, we report a method for the rapid and direct collection of differential kinetic data (direct measurement of rate) in flow by performing a series of instantaneous rate measurements on sequential small‐scale reactions. This technique decouples the time required to generate a full kinetic profile from the time required for a reaction to reach completion, enabling high throughput kinetic experimentation. In addition, comparison of kinetic profiles constructed at different residence times allows the robustness, or stability, of homogeneously catalysed reactions to be interrogated. This approach makes use of a segmented flow platform which was shown to quantitatively reproduce batch kinetic data. The proline mediated aldol reaction was chosen as a model reaction to perform a high throughput kinetic screen of 216 kinetic profiles in 90 hours, one every 25 minutes, which would have taken an estimated continuous 3500 hours in batch, an almost 40‐fold increase in experimental throughput matched by a corresponding reduction in material consumption.

## Graphical abstract

By performing a series of instantaneous rate experiments, flow chemistry can be used to collect differential kinetic profiles. The time taken to collect a profile becomes independent of the time it takes a reaction to reach completion. This effectively enables high throughput kinetic experimentation, where data can be collected faster than a reaction can run. The process can also probe catalytic stability, offering further mechanistic insights.

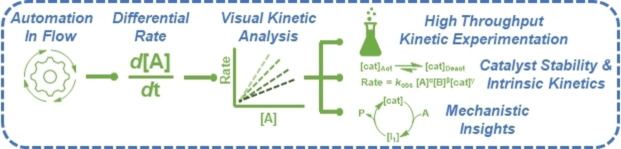

## Introduction

Kinetic investigation is central to the elucidation of reaction mechanisms and in understanding a chemical process. Only through such investigation can a reaction be rationally improved, leading to greener, safer, and more sustainable processes.^[1]^ While kinetic analysis has undergone a revitalisation in recent years, with the development of powerful and easy to use visual kinetic methods,^[2]^ the collection of experimental kinetic data can still prove to be a slow and laborious process. High throughput experimentation is employed routinely throughout industry and academia, where the use of multiwell plates delivers large volumes of single timepoint yield data, information on by‐product formation, and mass balance. These techniques have greatly increased chemists’ ability to screen and optimise reaction conditions,^[3]^ discover new reactivity,^[4]^ and generate datasets for exploration with artificial intelligence.^[5]^ Kinetic monitoring has been attempted in multiwell plates but does not realise the same increases in ease, speed, or throughput.^[6]^ The need for increased throughput in the capture of experimental kinetic data, reaction rates, and component orders remains unmet.

**Figure 1:**
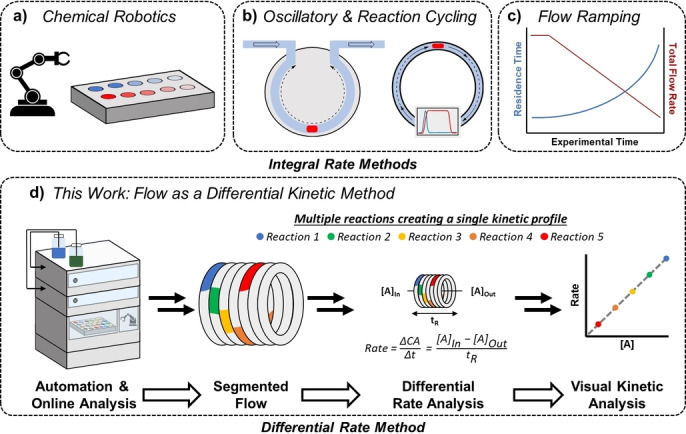
Kinetic data generated via: a) automation through robotic chemists; b) reaction cycling and oscillating flow reactors; c) flow‐ramping; d) this work.

The development of robotic chemists,^[7]^ or sampling and analysis by commercial or bespoke systems,^[8]^ is a burgeoning area of research and can minimise human labour through the automation of existing techniques and technologies (Figure 1a). Flow based approaches to kinetic monitoring have been able to attain some benefits of automation as well as a small increase in experimental throughput. Reaction cycling,^[9]^ and oscillating flow reactors,^[10]^ effectively trap a small‐scale reaction in flow and allow reaction progress to be monitored over time (Figure 1b). Flow manipulation, where reactor residence time is altered throughout an experiment using a transient flowrate,^[11]^ has proven a powerful approach amenable to coupling with kinetic modelling techniques,^[12]^ streamlining kinetic experimentation and analysis (Figure 1c).

A major drawback of flow chemistry is the requirement for rapid reactivity. Slow reactions, with residence times measured in even only tens of minutes and upwards, can be difficult to handle, requiring long residence times under which, above a certain level, the supposed benefits of flow are lost or become untenable. Although this can be alleviated somewhat by using oscillatory flow or reaction cycling techniques, an enormous amount of chemistry is simply unsuitable to conduct or monitor in flow.

All of the robotic and flow approaches described above (Figure 1), as well as the majority of common batch techniques, collect integral data (concentration over time). Fewer experimental techniques exist that collect differential data (the direct measurement of rate), the most common batch technique being isothermal calorimetry.^[13]^

Herein, we report a novel approach to collecting a full kinetic profile in a short, set time, regardless of how long the reaction actually takes to reach completion, without knowing how long the reaction will take a priori, and without the need for a pre‐defined kinetic model (Figure 1d). By treating flow chemistry as a differential kinetic technique, the rate of experimental data collection is greatly increased, representing a flow‐based method to high throughput kinetic experimentation.

## Results and Discussion

### Theoretical Approach

**Figure 2:**
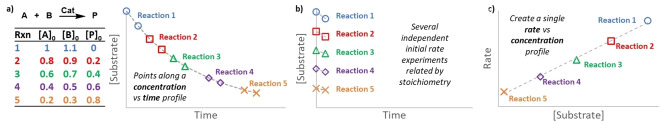
Relationship between independent initial rate experiments and a single kinetic profile through SPKA. Each colour represents a new reaction, a simulated point on a single kinetic profile collecting in either batch or flow: a) Concentration vs time plot for one experiment monitored over time; b) Concentration vs time plot for multiple independent reactions; c) Multiple reactions creating a single SPKA kinetic profile in a rate vs concentration plot.

Consider an integral kinetic profile of concentration against time (Figure 2a). Suppose that these points do not come from a single, parent, reaction but instead come from multiple independent reactions initiated at starting points along the parent reaction trajectory, similar to the same excess protocol of Blackmond's popular Reaction Progress Kinetic Analysis (RPKA).^[2a]^ Attempting to build a single integral kinetic profile from these independent reactions in a concentration vs time plot would be of no use (Figure 2b). However, by determining the instantaneous rate for each individual reaction, a single differential kinetic profile in a plot of rate vs concentration could be constructed (Figure 2c). Crucially, the time taken to collect this differential kinetic profile is now independent of the time the parent reaction takes to reach completion, meaning a full kinetic profile can be created without monitoring a reaction from start to finish. We have termed this approach Simulated Progress Kinetic Analysis (SPKA). SPKA is agnostic to the experimental approach used to collect data, this could be either multiple batch reactions, in standard round bottom flasks or a well plate, or sequential reaction segments in flow.

**Figure 3:**
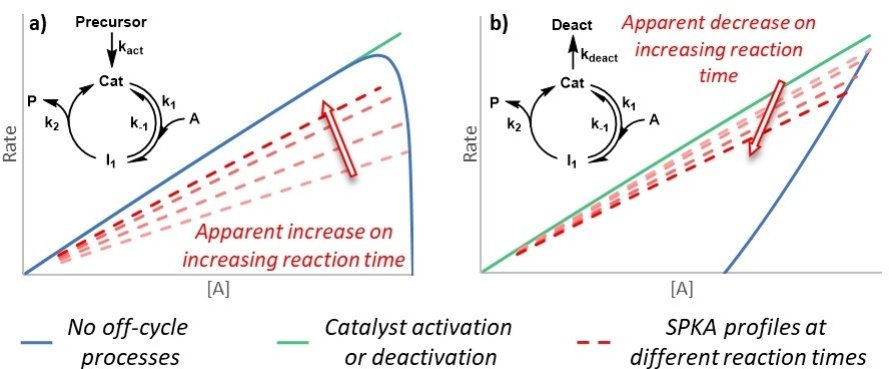
A simple, simulated, first order reaction demonstrating change in apparent rate by changing SPKA reaction times, used to probe for: a) catalyst activation resulting in an apparent increase in rate; b) catalyst deactivation resulting in an apparent decrease in rate (Supporting Information Section 1).

A perennially important issue in homogeneous catalysis is that of catalyst robustness, covering catalysts activation or deactivation as well as inhibition or acceleration. By comparing SPKA profiles created for the same parent reaction but collected over different instantaneous reaction times, SPKA can be used to probe catalyst robustness. If no off‐cycle processes are operating, then two such SPKA plots should be identical and will overlay (i.e., the different profiles will fall directly on top of one another). If, on increasing the reaction time, the apparent rate increases, then either catalyst activation or product acceleration are likely occurring; as more catalyst will have activated or more product will have formed, leading to higher conversions (Figure 3a). Conversely, if the apparent rate decreases, then either catalyst deactivation or product inhibition are likely to be taking place; as more catalyst is deactivated or product is formed over the time observed, leading to lower‐than‐expected conversions (Figure 3b). Even if an off‐cycle process is occurring, SPKA is based on an instantaneous rate approach and should largely negate any unwanted off‐cycle processes on the global kinetics, which are otherwise challenging to deconvolute.^[14]^

### A Platform to Collect Differential Kinetic Data in Flow

We reasoned that flow chemistry would be an ideal enabling technology to implement SPKA.^[15]^ The residence time and concentration of reagents going into the reactor coil are known, and if the concentration on exiting the coil can be instantaneously measured, then differential kinetic data can be collected.

**Figure 4:**
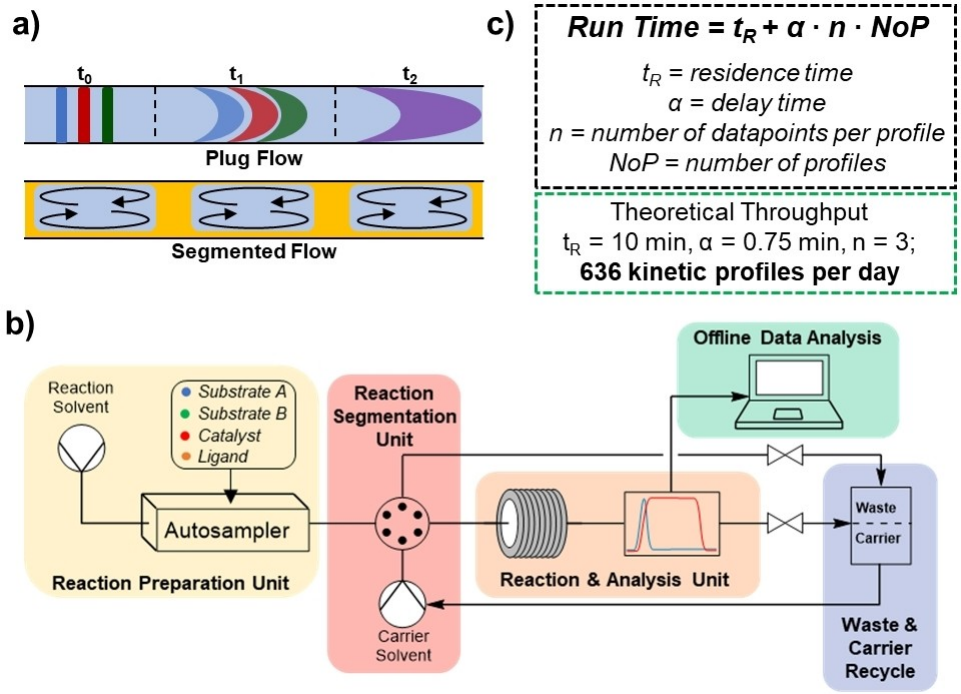
a) Representation of plug flow vs segmented flow; b) Idealised flow setup capable of performing SPKA, full description in Supporting Information Section 2; c) throughput calculations for constructing SPKA kinetic profiles in flow.

Ideally, tubular flow reactors display “plug flow” behaviour, with no mixing or diffusion axially in the direction of flow for each theoretical plug of fluid (Figure 4a). In reality, axial diffusion of reaction segments into the surrounding carrier solvent does occur, a fact used to great effect by Sach and co‐workers in their pioneering publication on high throughput experimentation in flow.^[16]^ However, as this leads to a continually decreasing reaction concentration, such a setup will clearly negatively affect any attempts at monitoring reaction kinetics. Segmented, or compartmentalised, flow is a biphasic flow regime in which one liquid phase, containing reactants, is divided into discrete segments by an immiscible carrier phase, typically either an inert gas or fluorous solvent. If using a fluorous carrier solvent, the only way to avoid contamination between segments is to ensure continuous preferential wetting of the tubing walls by the fluorous carrier. Practically, this rules out the use of steel tubing or small, bookending fluorous plugs.^[17]^ Each reaction segment is well mixed and completely isolated while still observing an identical reaction environment, making a segmented flow setup theoretically very well suited to the collection of differential kinetic data. Inspired by the work of Sach and co‐workers,[^16^, ^17^] we aimed to develop a segmented‐flow platform to realise high throughput kinetic experimentation (Figure 4b). Using a microfluidic setup results in major advantages of mixing times, temperature control, ease of automation, and materials savings.[^15^, ^18^] In this format, total throughput can be theorised, depending only on residence time, delay time (either for analysis or reaction preparation), the number of reactions per profile, and the number of profiles collected (Figure 4c). Fully optimised, a reasonable theoretical output of over 600 kinetic profiles could be generated in a single day (Supporting Information Section 2.6).

### Validation of SPKA

**Scheme 1:**
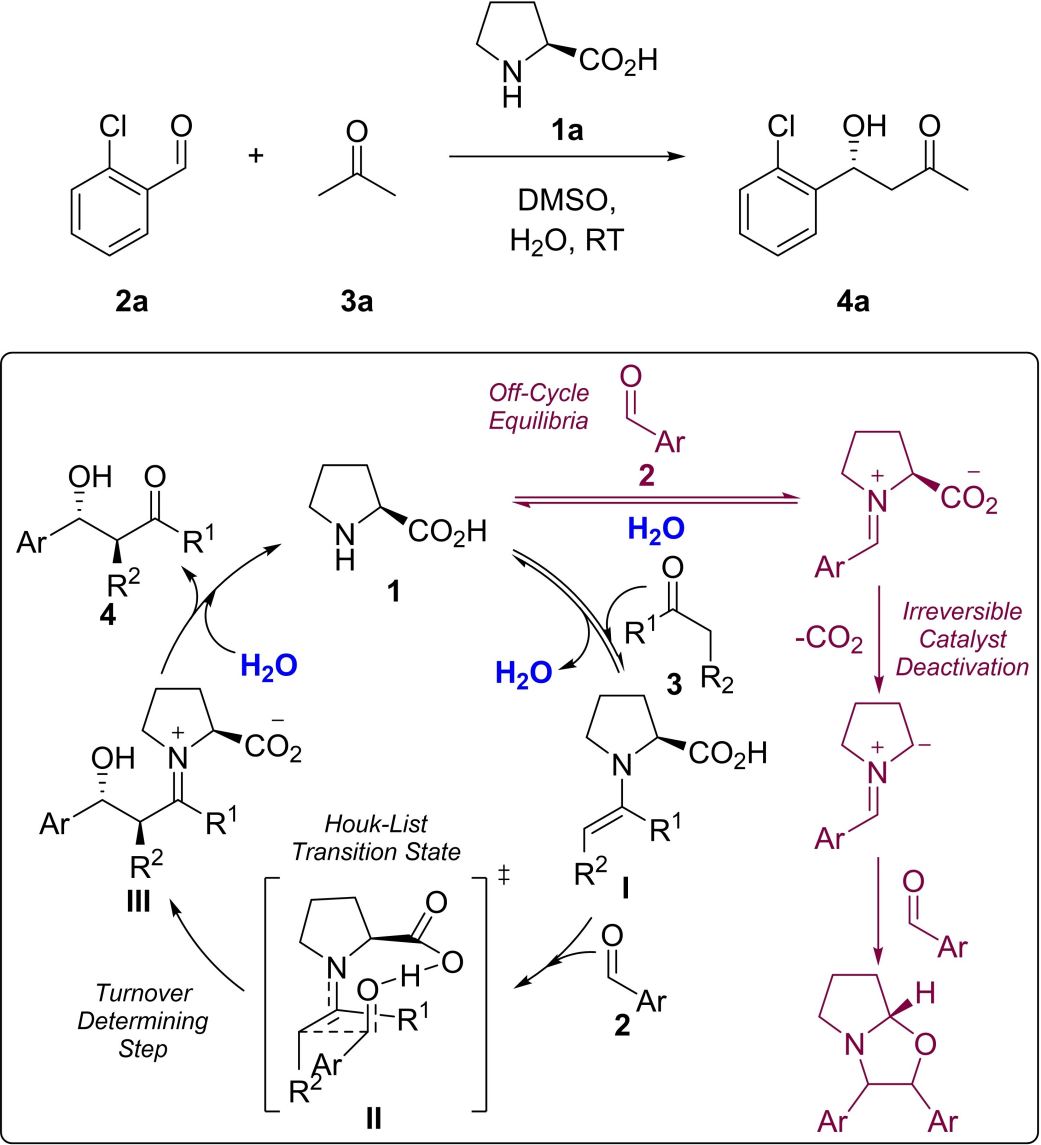
Proline mediated aldol reaction,^[19]^ showing the turnover determining step and irreversible off‐cycle deactivation processes as proposed by Armstrong and Blackmond.[^22^, ^24^]

To validate our platform, and SPKA, we chose to study the secondary amine mediated aldol reaction (Scheme 1).^[19]^ Asymmetric organocatalysis won List and MacMillan the 2021 Nobel Prize,[^19^, ^20^] with the field growing rapidly from their almost simultaneous publications at the turn of the century to a burgeoning and important area of research.^[21]^ The mechanism of the proline mediated aldol reaction was studied in detail by Blackmond and Armstrong, who found positive order kinetics in both aldehyde and ketone, implicating C−C bond formation as the rate limiting step,^[22]^ likely via the Houk‐List transition state which provides an almost quantitative model for the prediction of selectivity (Scheme 1).^[23]^ In a separate study, the same researchers elucidated the complex role of water in the catalytic cycle. While the addition of water slows the catalytic cycle, it also prevents catalyst deactivation through suppression of an off‐cycle pathway leading to the irreversible decarboxylation of proline.^[24]^

Despite being regarded by many as the archetypal organocatalyst, proline has well documented limitations. It is poorly soluble in most organic solvents, limiting solvent selection and often resulting in reaction slurries. It is a very sluggish catalyst, loadings of 20–30 mol % or above are common and, even then, reactions can still take 24 hours or more to reach completion. Very high loadings of carbonyl donor are often used to increase reaction rates, 20 vol % in the case of the original study by List and co‐workers.^[19]^ Its low activity is caused by off‐cycle deactivation processes, meaning diminishing returns on long reaction times. Poor solubility and long reaction times combine to make proline mediated systems a challenge to study by existing approaches to kinetic experimentation in flow.

**Figure 5:**
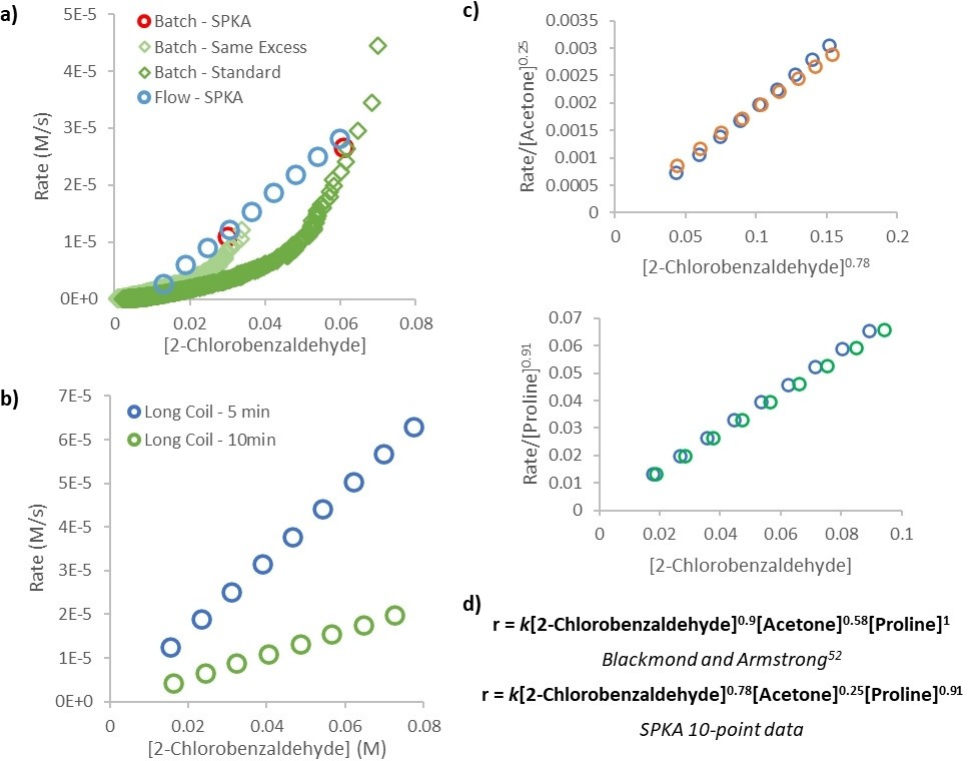
Platform validation: a) comparing batch and flow kinetic data. Batch same excess experiments (dark and light green diamonds), SPKA from batch data (red circles), SPKA from flow platform (blue circles); b) comparison of SPKA profiles collected at different residence times, lack of overlay clearly showing catalyst deactivation is occurring; c) RPKA different excess experiment using our flow platform; d) comparison of existing proline rate equation and that collected by our platform.

To collect an SPKA profile, ten reaction segments were created: a single 0 % conversion segment without catalyst to act as a reference point, and nine reaction segments of decreasing reagent concentration (equally spaced for a simulated 0–80 % conversion). These were pumped through a reactor coil for 11.4 minutes before the output substrate concentration was measured instantaneously by in‐line IR. The known inputs, and measured output, were used to create a 9‐point SPKA kinetic profile (Figure 5a, blue circles). Validating our platform required comparison against kinetics collected in batch (Figure 5a, green diamonds). A same excess experiment, the RPKA protocol for probing catalyst stability,^[2a]^ showed no overlay, meaning the catalytic cycle was not at steady state under our lower [H_2_O] conditions (Supporting Information Section 4). Calculating initial rates from our batch data, we were instead able to create a two‐point SPKA profile of identical residence time to that in flow (Figure 5a, red circles). Excellent agreement between the batch and flow SPKA profiles shows that our platform quantitatively matches the kinetic data collected in batch.

As the batch same excess experiments clearly show catalyst deactivation, we also wished to test whether this could be detected by a change in residence time SPKA experiment. The experiment was repeated with a decreased residence time (5.7 vs 11.4 minutes) and as expected, an apparently increased rate was observed at lower residence time (Figure 5b). Changes in flow rate can cause changes in mixing and flow regime. However, any physical origins of the difference in reactivity were ruled out through the observation of identical behaviour in reactor tubing of different lengths (Supporting Information Section 4.2).

To analyse the kinetic data produced by our platform, we chose to employ RPKA (Figure 5c).^[2a]^ RPKA uses differential rate vs concentration plots in a highly visual approach which will likely prove useful for rapid manual checks on large volumes of kinetic data. In addition, the different excess protocol of RPKA minimises the number of experiments required to probe a system, with only three experiments required to determine all the reagent orders in our reaction. In performing a different excess RPKA protocol, despite the mildly deactivating conditions used in our experiments, we found excellent agreement in component orders between our kinetic experiments and those reported by Blackmond and Armstrong (Figure 5d),[^22^, ^24^] particularly with partial orders in both reagents, lending additional confidence in our platform.

### High Throughput Kinetic Experimentation

We were able to further optimise the throughput of our platform by reducing the number of data points per profile from ten to five (one t_0_ and four reaction points), employing a 5‐minute residence time, and reducing the delay time between reaction plugs to 5 minutes, reaching the physical limitations of our current hardware. Under these conditions, a single kinetic profile could be collected in just 25 minutes, regardless of how long the reaction actually takes, using a total reaction volume of only 0.75 mL. Run continuously, this would equate to 57 kinetic profiles every 24 hours. Custom Python scripts were written to handle this large volume of data, creating SPKA profiles from IR data and finding reaction orders (Supporting Information Section 3).

**Scheme 2:**
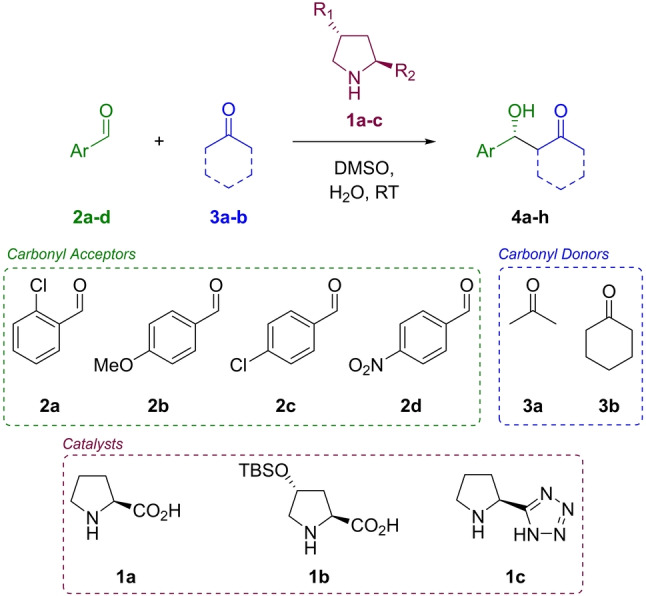
Scope of kinetic screen (Supporting Information Section 5).

With an optimised platform in hand, the ability to collect quantitative rate data confirmed, and with good agreement between our component orders and those in the literature, we wished to perform a kinetic and mechanistic screen of the proline mediated aldol reaction. We chose to perform a full kinetic investigation of four carbonyl acceptors, two carbonyl donors, and three catalysts (Scheme 2), although our substrate choices were constrained by the practical limitations of our platform (Supporting Information Section 5.5).

Derivatives and analogues of proline (**1 a**) have been developed in attempts to address its limitations, but few have been contrasted and compared experimentally in terms of absolute reaction rates or reagent orders. Hayashi and co‐workers developed siloxy proline analogue **1 b**, first reporting it as a more soluble, and more active, analogue of proline in the α‐aminoxylation reaction,^[25]^ later showing the catalyst to be active in the aldol reaction to loadings as low as 1 % in the presence of 300 mol % water.^[26]^ Proline mediated α‐aminoxylation has a different turnover determining step than the aldol reaction,^[27]^ and so we were curious whether **1 b** would still accelerate the aldol reaction relative to proline in organic solvents. The use of proline tetrazole (**1 c**) as an organocatalyst was reported almost simultaneously by the groups of Ley,^[28]^ Arvidsson,^[29]^ and Yamamoto.^[30]^ Tetrazole is a carboxylic acid isostere, a common replacement in medicinal chemistry, and one which greatly improves the solubility of proline tetrazole (**1 c**) to a range of solvents other than dimethyl sulfoxide (DMSO). The activity of proline tetrazole (**1 c**) is generally reported as similar to or greater than proline (**1 a**) in a number of transformations.^[29]^ In addition to reported differences in activity, we wondered whether we might potentially observe a shift in turnover determining step under proline tetrazole (**1 c**) indicated by a change in the kinetics.

Our substrate scope (Scheme 2) results in a total of 24 possible reagent combinations. Each combination requires three reactions to determine the order in each component, and we chose to run each system in triplicate to ensure the quality of our data. This resulted in a total of 216 experiments. It is impossible to state, a priori, how long this screening would take in batch, the time required for any of the reactions to reach completion cannot be known until they are run, and this represents one of the biggest advantages of SPKA. If all reactions took a similar time as the batch reaction of 2‐chlorobenzaldehyde (**2 a**), acetone (**3 a**), and proline (**1 a**) under the conditions chosen for our study (16 hours) sequential reaction monitoring would take almost 3500 hours, over four months of continuous monitoring. Using our approach, this full factorial, in triplicate screen would take a fixed 90 hours.

For every substrate combination, except 2‐chlorobenzaldehyde (**2 a**), proline (**1 a**) displayed a higher rate than either derivatised analogue, with this particularly pronounced for 4‐nitrobenzaldehyde (**2 d**) (Table 1). These results suggest that reported increased rates observed for proline‐OTBS (**1 b**) in the aldol reaction,[^25^, ^26^] are likely related more to increased solubility of the catalyst than inherently increased reactivity.

**Table 1:**
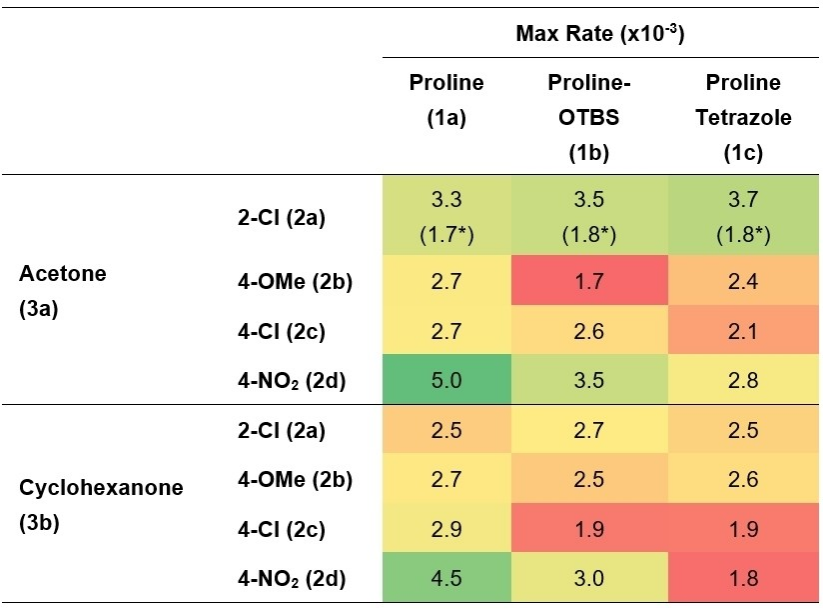
Heatmap of maximum reaction rates (Supporting Information Section 5.7).

*Different residence time experiment (t_r_ =5.7 min)

Similar partial orders in carbonyl donor and acceptor were observed for all systems, suggesting no change in mechanism on change in catalyst or substrate (Table 2). In agreement with Blackmond and Armstrong,^[22]^ this suggests that C−C bond formation is turnover limiting and that free catalyst (**1**) and enamine (**I**) share the role of turnover determining intermediate, the fleeting iminium species assumed to immediately collapse into detectable enamine (**I**) (Scheme 1).^[31]^ Proline tetrazole (**1 c**) has slightly larger orders in carbonyl donor than either proline (**1 a**) or proline‐OTBS (**1 b**), more pronounced with cyclohexanone (**3 b**). A change in the magnitude of the order relates to a shift in the relative abundance of turnover determining intermediate on the catalytic cycle. Mechanistically, this suggests a shift towards free catalyst (**1**) and away from enamine (**I**) as the dominant turnover determining intermediate and means the concentration of carbonyl donor has a larger effect on the reaction rate than for the other catalysts. This might explain why other reports in the literature suggest that proline tetrazole (**1 c**) is faster than proline, as these typically employ significantly higher loadings of carbonyl donor, up to 20 vol %, which would have a greater effect on proline tetrazole (**1 c**) than either proline (**1 a**) or proline‐OTBS (**1 b**).^[29b]^

**Table 2:**
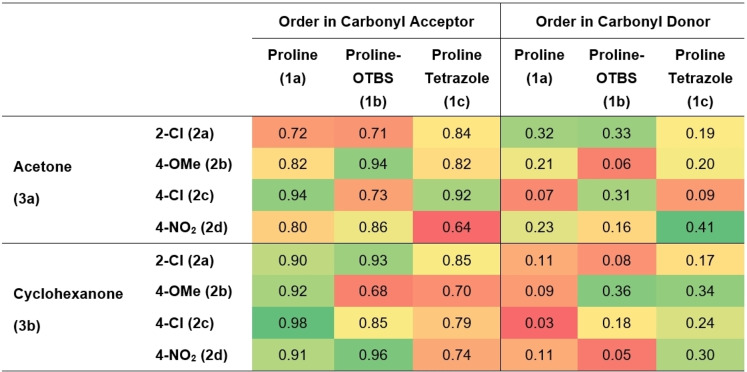
**H**eatmap, divided between carbonyl acceptor and donor, showing orders in carbonyl acceptor and donor (Supporting Information Section 5).

Only a handful of reactions displayed catalyst order close to one, with the majority sitting closer to 0.5 (Supporting Information Section 5.7). We wondered whether this order of less than one was a consequence of a lack of stability in the catalytic cycle. Indeed, no overlay was observed for either proline‐OTBS (**1 b**) or proline tetrazole (**1 c**) in a change in residence time experiment (Supporting Information Section 5.6). Proline undergoes deactivation through irreversible decarboxylation after condensation with a carbonyl acceptor (Scheme 1) and it is likely that proline‐OTBS (**1 b**) follows an identical deactivation pathway.^[24]^ Indeed, mixing proline‐OTBS (**1 b**) with an aldehyde in DMSO‐d6 resulted in the appearance of characteristic NMR signals for the decarboxylation‐cycloaddition product. Proline tetrazole (**1 c**) cannot undergo decarboxylation and so an alternative mechanistic explanation must be sought. Zotova and Blackmond briefly investigated the proline tetrazole (**1 c**) mediated aldol reaction in batch, finding, as we do, a lack of overlay in a same excess experiment.^[32]^ Further experimentation led the authors to suggest that the formation of a double aldol addition product was occurring, and it is this unwanted, and unaccounted for, consumption of the carbonyl acceptor that is causing a lack of same excess overlay rather than deactivation of proline tetrazole (**1 c**). These results highlight the potential, and significance, for catalyst deactivation, or other unwanted off‐cycle processes, to be probed early in process development as part of a screening process using SPKA.

## Conclusion

We have demonstrated that flow chemistry can be employed as a differential kinetic method to realise high throughput kinetic experimentation. The instantaneous rate data generated in this manner can be transformed into a single kinetic profile through a method we have termed SPKA, allowing for the use of powerful and popular visual kinetic analysis techniques. SPKA offers a number of advantages as a differential kinetic method. An SPKA profile takes a fixed and constant time to collect, faster than the actual time a reaction takes to reach completion. A change in residence time protocol was also shown to be able to probe the robustness of a catalytic cycle. The approach was implemented in a segmented flow platform which, although not without practical limitations in its current format, was able to quantitatively reproduce batch kinetic data of a model proline mediated aldol reaction. A kinetic screen of the aldol reaction was performed, consisting of three reactions for each of 24 possible reagent combinations all performed in triplicate. The resulting 216 experiments took a fixed 90 hours to complete, as opposed to an estimated 3500 hours if monitored in batch. The process can be fully automated and run as easily as most walk‐up analytical techniques, even by non‐experts in kinetics. 57 Kinetic profiles, or 285 individual reactions, in a 24‐hour period represents the physical limit of the hardware used in this study but not the maximum throughput of the approach which, with the right equipment, could reach >600 kinetic profiles per day. As high throughput experimentation revolutionised the rapid screening of compound libraries and reaction conditions, we believe high throughput kinetic experimentation, powered by SPKA, could have a similar effect on mechanistic study in academic and industrial laboratories.

## Supporting Information

The authors have cited additional references within the Supporting Information. All Python scripts are available free of charge at GitHub: https://github.com/pdingwall/htke. The data that support the findings of this study are openly available in the PURE repository at doi.org/10.17034/f5242e69‐5f69‐4267‐8652‐7cebb26a4cd2

## Conflict of interest

The authors declare no conflict of interest.

1

## Supporting information

As a service to our authors and readers, this journal provides supporting information supplied by the authors. Such materials are peer reviewed and may be re‐organized for online delivery, but are not copy‐edited or typeset. Technical support issues arising from supporting information (other than missing files) should be addressed to the authors.

Supporting Information

## Data Availability

The data that support the findings of this study are openly available in PURE at https://doi.org/10.17034/f5242e69‐5f69‐4267‐8652‐7cebb26a4cd2, reference number 100.
