# Supplementary material for: Enabling High Throughput Kinetic Experimentation by Using Flow as a Differential Kinetic Technique
Source: Angew Chem Int Ed Engl. 2023 Dec 22;63(5):e202318146. doi: 10.1002/anie.202318146 (PMC10952970; doi:10.1002/anie.202318146)
Supplement: Supplementary file 1 — Supporting Information [file ANIE-63-0-s001.pdf]

Angewandte Chemie
Eine Zeitschrift der Gesellschaft Deutscher Chemiker

Supporting Information

Enabling High Throughput Kinetic Experimentation by Using Flow as a Differential Kinetic Technique

*G. Lennon, P. Dingwall**

Contents

1 SPKA Theory 3
1.1 Probing Catalyst Stability 3
2 Platform Design 4
2.1 General Experimental Method 4
2.2 Reaction Preparation 5
2.3 Carrier Phase Wetting Preferences and Segmentation 6
2.4 Plug Isolation 7
2.5 Plug Mixing in Segmented Flow 7
2.6 Maximum Theoretical Throughput 8
3 Data Processing 10
3.1 Peak Picking and Error Analysis 10
3.2 Linear Correction 12
4 Platform Validation 14
4.1 SPKA Flow vs Batch Data 14
4.2 Residence Time: Flow Rate vs Coil Length Studies 15
4.3 Non-Segmented Flow 16
5 High Throughput Kinetic Experimentation 19
5.1 Conditions 19
5.2 Catalyst Stock Solutions 19
5.3 10 vs 5-point data 19
5.4 Repeatability 20
5.5 Practical Limitations and Unsuitable Systems 21
5.6 Proline-OTBS (1b) and Proline Tetrazole (1c) Different Residence Time Study 22
5.7 Data Analysis 24
5.7.1 3D Plots 24
5.7.2 Carbonyl Donor (Rate) 24
5.7.3 Carbonyl Acceptor (Rate) 26

5.7.4 Catalyst (Rate) 27
5.8 Reagent Order Analysis 28
6 References 30

1 SPKA Theory

1.1 Probing Catalyst Stability

A simplified, single intermediate catalytic cycle displaying first order in substrate was used with either sow catalyst activation or deactivation (Figure S1), data was simulated using COPASI.[1] Model parameters are found in Table S1.

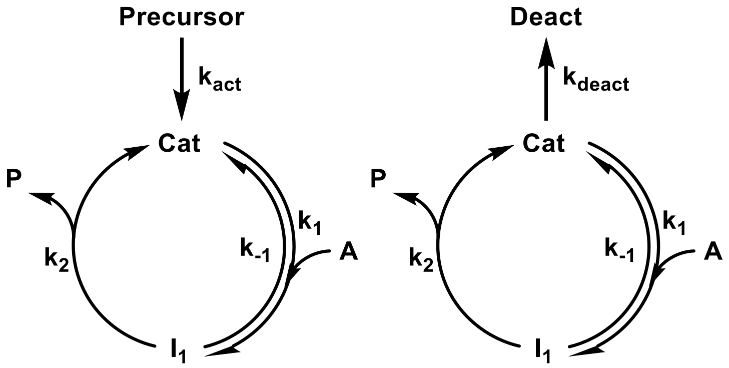

Figure S1: Simple, single intermediate, first order catalytic cycles including catalyst activation and deactivation

Table S1: COPASI model parameters and initial conditions

Parameter	Value
Compartment	1 mL
$[A]_0$	1 M
$[Precursor]_0/[Cat]_0$	0.01 M
k_1	0.1
k_{-1}	1
k_2	1
k_{act}/k_{deact}	0.001

SPKA profiles were created with residence times of 2, 4, 6, and 8 minutes from conversions of 10-100% in 10% intervals.

2 Platform Design

The platform designed for this study is based around an Agilent 1100 HPLC system (Figure S2). The reaction preparation unit comprises of an Agilent 1100 G1311A quaternary pump and G1329A autosampler with G1330B autosampler thermostat. The reaction segmentation unit comprises of an Agilent G1158A 2/6-valve and G1311A quaternary pump. The reaction and analysis unit comprises of a reactor coil, 314.5 cm of 1.14 mm ID tubing (Adtech TW18, PTFE), wound around a container full of water situated on a hotplate with the water held at a constant 29°C, and a Mettler Toledo FlowIR, with a 50 µL head, sampling at a frequency of 15 s. The system is controlled by Agilent's OpenLab software (Acquisition Build 2.3.7.5) and connects to the system through a G1314A VWD, although this is not used for experimentation. Data analysis was conducted using custom python scripts, described below.

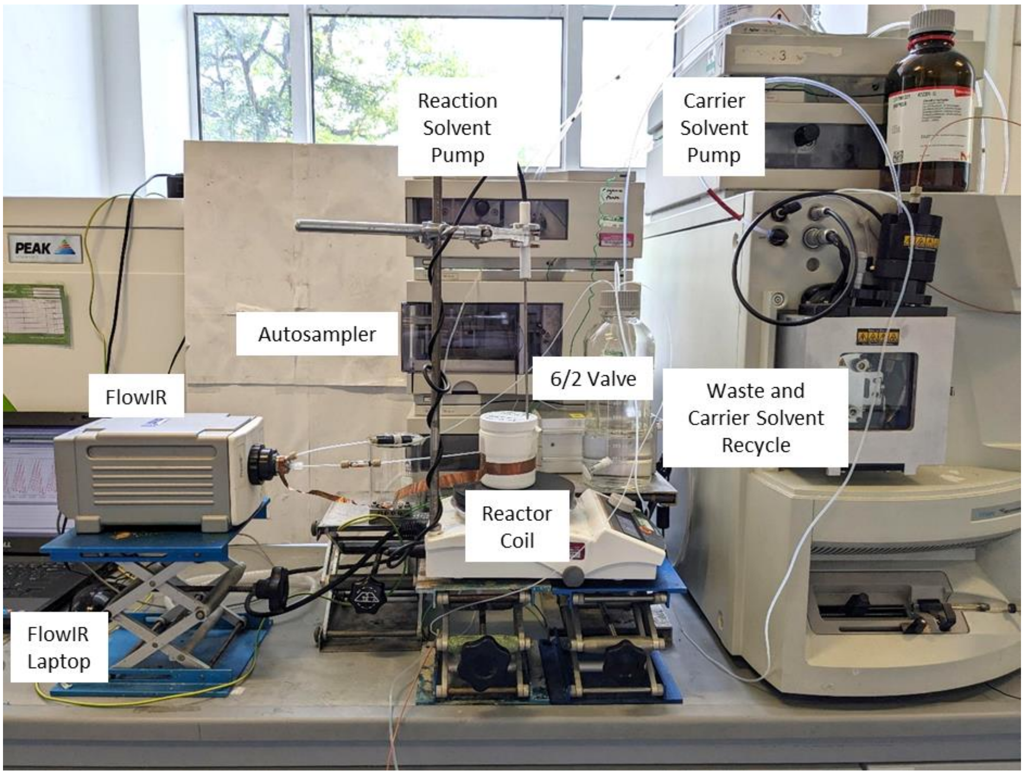

Figure S2: Photograph of the platform

2.1 General Experimental Method

The general method follows the steps outlined in Table S2.

Table S2: General experimental method

Step	Description
1	Perform a wash cycle: Wash the needle in a vial of water then a vial of DMSO (aq.). A plug of 80 µL of DMSO (aq.) reaction solvent was created and introduced to the reactor coil to wash the FlowIR window.
2	A reaction plug of desired concentrations was created from the required volume of each stock solution. This was performed in the order: make up DMSO (aq.), ketone, aldehyde, catalyst.
3	The autosampler injects the reaction plug, which flows a short distance to the standalone 2/6-valve. The 2/6-valve switches, allowing the reaction plug to enter the reactor coil. After a 15 second delay, the 2/6-valve switches back, isolating a reaction-plug of 150 µL in a moving flow of carrier solvent.
4	Repeat.

2.2 Reaction Preparation

A single HTKE run consisted of a screen of one catalyst and one aldehyde with two ketones. Each run was conducted in triplicate, resulting in 90 reaction plugs creating 18 kinetic profiles over 7.5 hours, allowing two systems to be run in a single working day.

Stock solutions of each reaction component (ketones, aldehyde, catalyst, make-up solvent, and make-up catalyst stock) were made up in HPLC vials (Supelco 9mm 2 mL P/N: 29378-U) and were placed in the autosampler which was held at a constant 20 °C. Stock solution locations in the autosampler are listed in Table S3.

Table S3: Stock solution layout in autosampler

Position	Stock
1	Make up catalyst solution
2	Catalyst
4	Aldehyde
5	Acetone
6	Ketone
7	Make up DMSO (aq.) solvent
8	DMSO (aq.) wash
12	Catalyst – for different excess
15	Acetone – for different excess
16	Ketone – for different excess
98	Water
99	DMSO (aq.) wash
100	DMSO (aq.) wash

Reaction plugs were created using the Sample Prep Injector Programme in Agilent OpenLab. Each reaction plug was created to a constant 27 µL, which, due to varying volumes of each stock solution, was upheld using a make-up vial of DMSO (aq.). The initial plug for an SPKA run was the t_0, Figure S3, which was created identically to the "0% SPKA" plug, but with the catalyst component replaced with blank solvent, Figure S4. A unique injector programme is required for every SPKA conversion point.

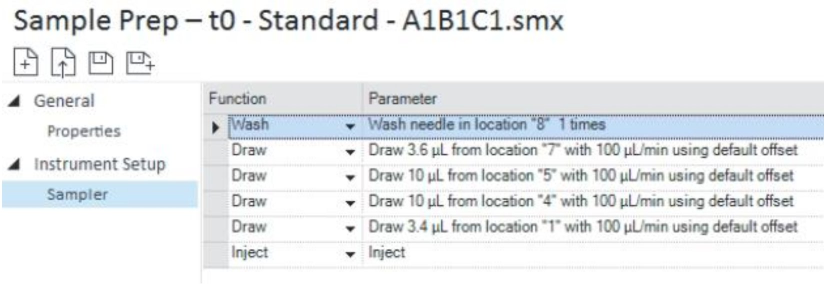

Figure S3: Example t_0 injector programme

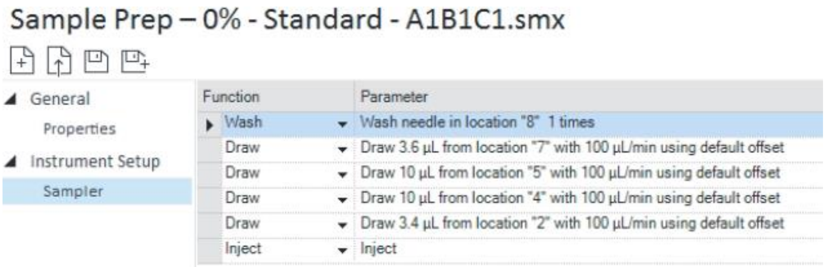

Sample Prep – 0% - Standard - A1B1C1.smx

Function	Parameter
Wash	Wash needle in location "8" 1 times
Draw	Draw 3.6 µL from location "7" with 100 µL/min using default offset
Draw	Draw 10 µL from location "5" with 100 µL/min using default offset
Draw	Draw 10 µL from location "4" with 100 µL/min using default offset
Draw	Draw 3.4 µL from location "2" with 100 µL/min using default offset
Inject	Inject

Figure S4: Example reaction plug injector programme

The injector programmes were run using a standard OpenLab sequence table (Figure S5). Each reaction plug alternates with a wash plug, to wash both the outside of the autosampler needle and the FlowIR window.

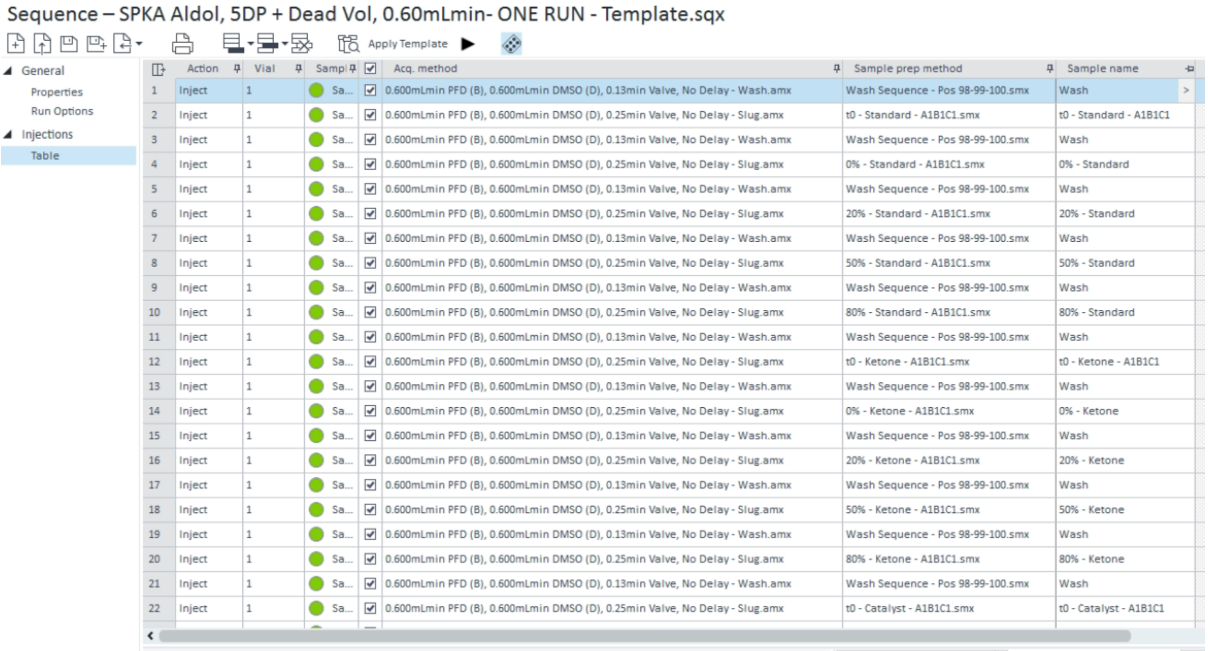

Sequence – SPKA Aldol, 5DP + Dead Vol, 0.60mLmin- ONE RUN - Template.sqx

	Action	Vial	Sampl		Acq. method	Sample prep method	Sample name
1	Inject	1	Sa...	☑	0.600mLmin PFD (B), 0.600mLmin DMSO (D), 0.13min Valve, No Delay - Wash.amx	Wash Sequence - Pos 98-99-100.smx	Wash
2	Inject	1	Sa...	☑	0.600mLmin PFD (B), 0.600mLmin DMSO (D), 0.25min Valve, No Delay - Slug.amx	t0 - Standard - A1B1C1.smx	t0 - Standard - A1B1C1
3	Inject	1	Sa...	☑	0.600mLmin PFD (B), 0.600mLmin DMSO (D), 0.13min Valve, No Delay - Wash.amx	Wash Sequence - Pos 98-99-100.smx	Wash
4	Inject	1	Sa...	☑	0.600mLmin PFD (B), 0.600mLmin DMSO (D), 0.25min Valve, No Delay - Slug.amx	0% - Standard - A1B1C1.smx	0% - Standard
5	Inject	1	Sa...	☑	0.600mLmin PFD (B), 0.600mLmin DMSO (D), 0.13min Valve, No Delay - Wash.amx	Wash Sequence - Pos 98-99-100.smx	Wash
6	Inject	1	Sa...	☑	0.600mLmin PFD (B), 0.600mLmin DMSO (D), 0.25min Valve, No Delay - Slug.amx	20% - Standard - A1B1C1.smx	20% - Standard
7	Inject	1	Sa...	☑	0.600mLmin PFD (B), 0.600mLmin DMSO (D), 0.13min Valve, No Delay - Wash.amx	Wash Sequence - Pos 98-99-100.smx	Wash
8	Inject	1	Sa...	☑	0.600mLmin PFD (B), 0.600mLmin DMSO (D), 0.25min Valve, No Delay - Slug.amx	50% - Standard - A1B1C1.smx	50% - Standard
9	Inject	1	Sa...	☑	0.600mLmin PFD (B), 0.600mLmin DMSO (D), 0.13min Valve, No Delay - Wash.amx	Wash Sequence - Pos 98-99-100.smx	Wash
10	Inject	1	Sa...	☑	0.600mLmin PFD (B), 0.600mLmin DMSO (D), 0.25min Valve, No Delay - Slug.amx	80% - Standard - A1B1C1.smx	80% - Standard
11	Inject	1	Sa...	☑	0.600mLmin PFD (B), 0.600mLmin DMSO (D), 0.13min Valve, No Delay - Wash.amx	Wash Sequence - Pos 98-99-100.smx	Wash
12	Inject	1	Sa...	☑	0.600mLmin PFD (B), 0.600mLmin DMSO (D), 0.25min Valve, No Delay - Slug.amx	t0 - Ketone - A1B1C1.smx	t0 - Ketone - A1B1C1
13	Inject	1	Sa...	☑	0.600mLmin PFD (B), 0.600mLmin DMSO (D), 0.13min Valve, No Delay - Wash.amx	Wash Sequence - Pos 98-99-100.smx	Wash
14	Inject	1	Sa...	☑	0.600mLmin PFD (B), 0.600mLmin DMSO (D), 0.25min Valve, No Delay - Slug.amx	0% - Ketone - A1B1C1.smx	0% - Ketone
15	Inject	1	Sa...	☑	0.600mLmin PFD (B), 0.600mLmin DMSO (D), 0.13min Valve, No Delay - Wash.amx	Wash Sequence - Pos 98-99-100.smx	Wash
16	Inject	1	Sa...	☑	0.600mLmin PFD (B), 0.600mLmin DMSO (D), 0.25min Valve, No Delay - Slug.amx	20% - Ketone - A1B1C1.smx	20% - Ketone
17	Inject	1	Sa...	☑	0.600mLmin PFD (B), 0.600mLmin DMSO (D), 0.13min Valve, No Delay - Wash.amx	Wash Sequence - Pos 98-99-100.smx	Wash
18	Inject	1	Sa...	☑	0.600mLmin PFD (B), 0.600mLmin DMSO (D), 0.25min Valve, No Delay - Slug.amx	50% - Ketone - A1B1C1.smx	50% - Ketone
19	Inject	1	Sa...	☑	0.600mLmin PFD (B), 0.600mLmin DMSO (D), 0.13min Valve, No Delay - Wash.amx	Wash Sequence - Pos 98-99-100.smx	Wash
20	Inject	1	Sa...	☑	0.600mLmin PFD (B), 0.600mLmin DMSO (D), 0.25min Valve, No Delay - Slug.amx	80% - Ketone - A1B1C1.smx	80% - Ketone
21	Inject	1	Sa...	☑	0.600mLmin PFD (B), 0.600mLmin DMSO (D), 0.13min Valve, No Delay - Wash.amx	Wash Sequence - Pos 98-99-100.smx	Wash
22	Inject	1	Sa...	☑	0.600mLmin PFD (B), 0.600mLmin DMSO (D), 0.25min Valve, No Delay - Slug.amx	t0 - Catalyst - A1B1C1.smx	t0 - Catalyst - A1B1C1

Figure S5: Example sequence table

Example injector programmes, methods, and sequences used for the high throughput kinetic runs are included in the PURE data repository and can be downloaded and used for free.

2.3 Carrier Phase Wetting Preferences and Segmentation

As is shown in great detail by Blackmond, Sach and co-workers,[2] the wetting preference of the tubing and carrier solvent is of great importance in compartmentalised flow systems. For perfluorodecalin to preferentially wet the tubing, the tubing must be made from fluorous polymer, any change of tubing material, i.e., steel, inverts the wetting preference and the reaction solvent preferentially coats the tubing instead. The material used internally to the G1329A autosampler is almost exclusively steel, an while some could be replaced with PTFE tubing, the sampler needle could not. As a result, we found it impossible to introduce perfluorodecalin to the autosampler and maintain stable reaction plugs. To solve this issue, only reaction solvent flows through the autosampler and segmentation occurs at a separate 2/6-valve (Figure S6).

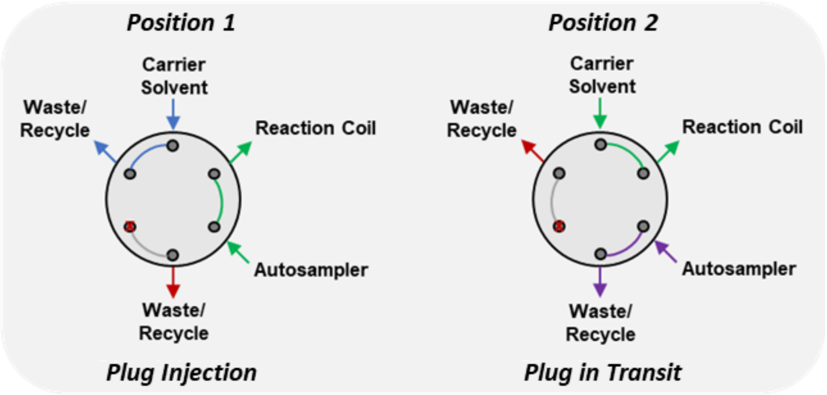

Figure S6: Flow segmentation, and plug creation, at 2/6-valve.

The volume of the plug is determined by the flowrate of the reaction solvent and the length of time the valve stays in position 1. For the five-minute residence time used for the high throughput kinetic experiments, a reaction solvent flowrate of 0.6 mL min^{-1} and valve time of 15 s were used to create 150 µL plugs. The flowrate of the carrier solvent was matched to 0.6 mL min^{-1} to maintain a smooth velocity throughout the coil on change of valve position.

2.4 Plug Isolation

A blue dye was used to demonstrate plug isolation (Figure S7). As can be seen, no blue dye is present in the wash slug, which is just visible near the top of the reactor coil. A time-lapse video (one picture taken every 2 seconds, one frame every 0.1 seconds) is available in the data repository showing the operation of plug flow.

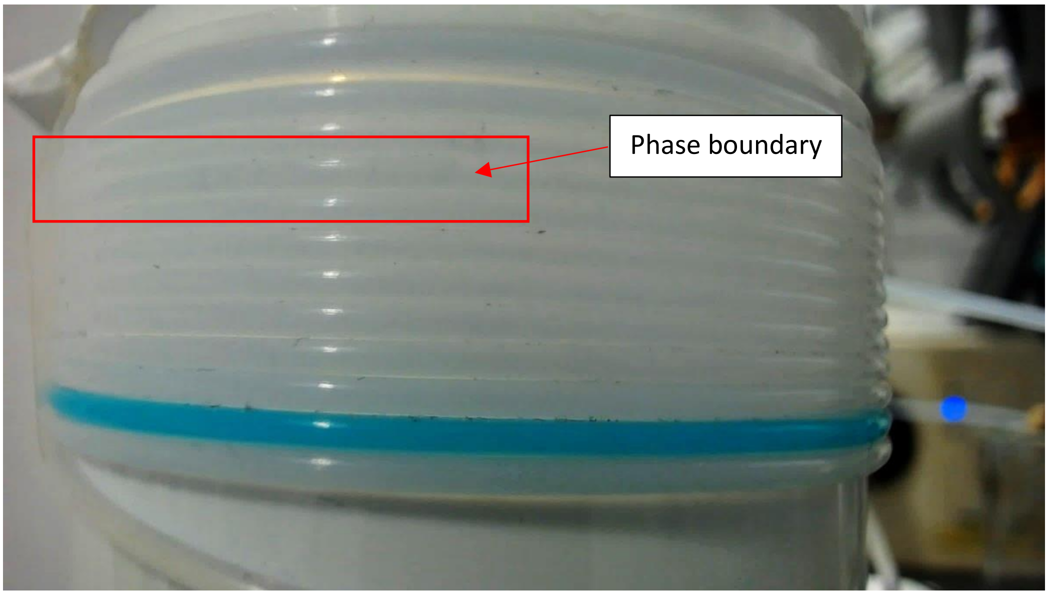

Figure S7: Photograph showing a 150 µL plug with blue dye (bottom) and a clear wash slug (top – phase boundary just visible) demonstrating no carryover between plugs

2.5 Plug Mixing in Segmented Flow

Mixing of components within the plug was examined. If uniform mixing of all reaction components is achieved, the ratio of analytes should remain constant across the length of the reaction plug. Conversely, if poor mixing occurs, certain analytes will be found to dominate at different positions

along the length of the reaction plug in line with their order of addition during reaction segment preparation (Figure S8).

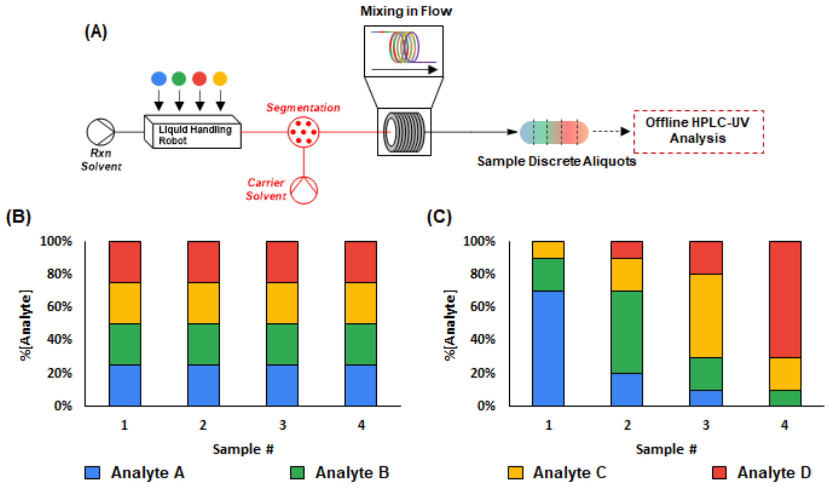

Figure S8: Graphical description of: A) Reactor design used to study mixing in segmented plugs; B) analyte ratio under ideal mixing conditions; C) analyte ratio under non-ideal mixing conditions.

Four-component reaction plugs were prepared, using stock solutions of UV-active analytes: biphenyl, benzophenone, benzaldehyde, and propiophenone. Stock solution concentrations were tailored so the UV-response of each analyte was approximately equal at fixed detection wavelength (254 nm). Each 150 µL plug was partitioned into 5 samples and analysed by offline HPLC-UV. Uniform analyte distributions were determined across the plugs at various reactor lengths, denoting facile mixing under the experimental conditions (Figure S9).

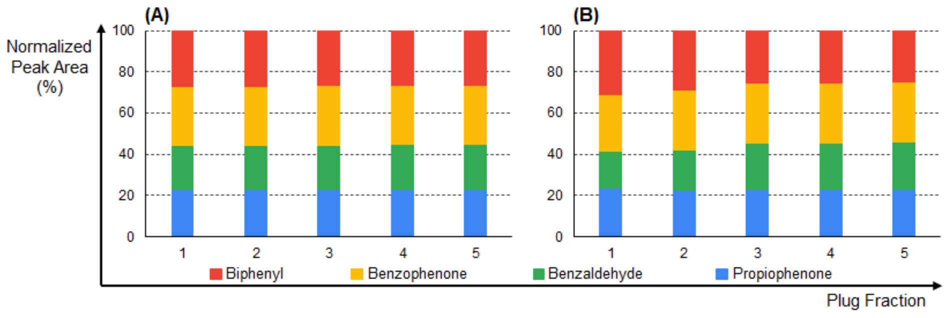

Figure S9: Mixing studies using a PTFE reactor coil of dimensions: A) 151.2 cm x 1.14 mm (L x ID); B) 49.3 cm x 1.14 mm (L x ID).

2.6 Maximum Theoretical Throughput

The maximum throughput for a flow based SPKA platform can be simply determined. If multiple reaction plugs are present simultaneously in the reaction coil, then the time taken to collect a kinetic profile is determined by Equation S1, where t_R is the residence or reaction time, α is the delay time between plugs, n is the number of datapoints per profile, and NoP is number of profiles to be collected.

$$Run\ Time = t_R + \alpha \cdot n \cdot NoP \qquad \textbf{Equation S1}$$

If the run time is taken to be 24 hours and the residence time 10 minutes, then the effect of the delay time and number of points per reaction on the theoretical number of profiles per day can be examined (Figure S10). A delay time of 45s (0.75 min) has been shown feasible if two low resolution mass spec units are used in parallel.[3]

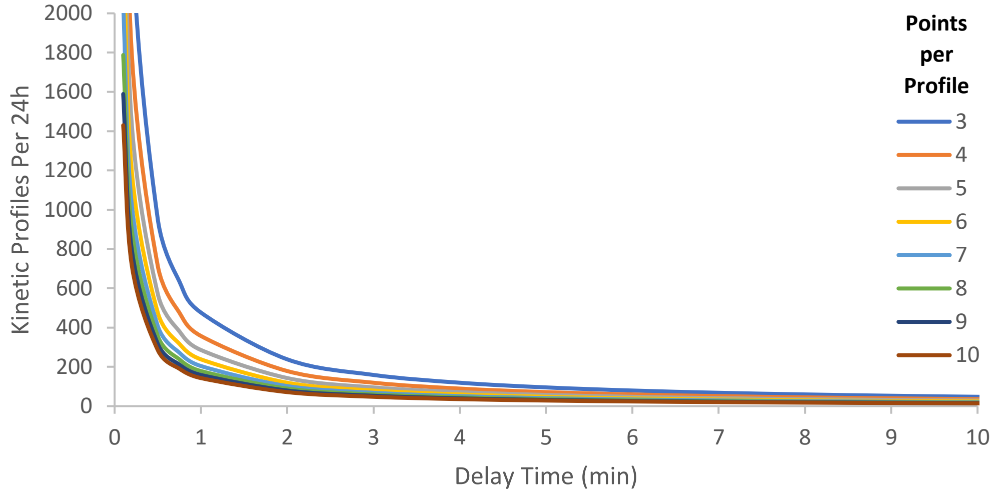

Delay Time (min)	Points per Profiles 3	4	5	6	7	8	9	10
0.1	4767	3575	2860	2383	2043	1788	1589	1430
0.2	2383	1788	1430	1192	1021	894	794	715
0.5	953	715	572	477	409	358	318	286
0.75	636	477	381	318	272	238	212	191
1	477	358	286	238	204	179	159	143
2	238	179	143	119	102	89	79	72
3	159	119	95	79	68	60	53	48
4	119	89	72	60	51	45	40	36
5	95	72	57	48	41	36	32	29
6	79	60	48	40	34	30	26	24
7	68	51	41	34	29	26	23	20
8	60	45	36	30	26	22	20	18
9	53	40	32	26	23	20	18	16
10	48	36	29	24	20	18	16	14

Figure S10: Maximum theoretical throughput for a flow based SPKA setup based on Equation S1.

3 Data Processing

Raw data was processed using a custom python script which can be found at GitHub: https://github.com/pdingwall/htke. The associated custom functions in the various .py files are heavily commented to aid understanding. A walkthrough of the processing method can be found in the jupyter notebook in the examples folder on GitHub.

3.1 Peak Picking and Error Analysis

The output IR data for SPKA consists of many peaks over time. In this experiment, a typical 't0' plug for the aldol reaction (SPKA conversion of 0, initial concentrations at their highest, minus catalyst so reaction cannot occur) was created and repeated 62 times (Figure S11) with a residence time of 10 minutes.

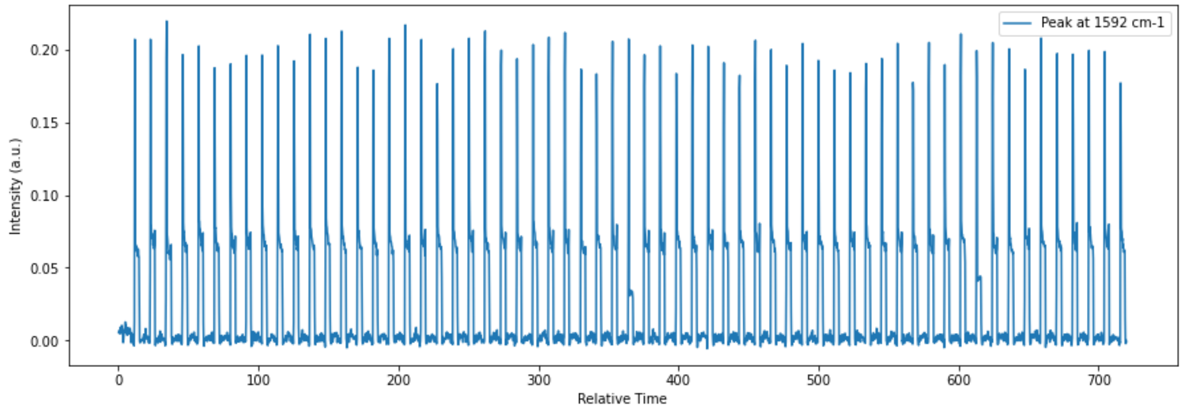

Figure S11: IR plot of Intensity vs Relative Time, this experiment contains the same plug makeup repeated 62 times.

Different approaches can be used to quantify the property of each peak. Scipy.signal.find_peaks allows the determination of the peak height or prominence. Alternatively, the area under each peak can be integrated to find an experimental area, or an ideal gaussian distribution can be best fit to the data and then integrated (Figure S12). In segmented flow, IR peaks are not an ideal gaussian shape, due to accumulation on the IR window, this results in poor performance of the height property in reaction data; therefore, this property was not used.

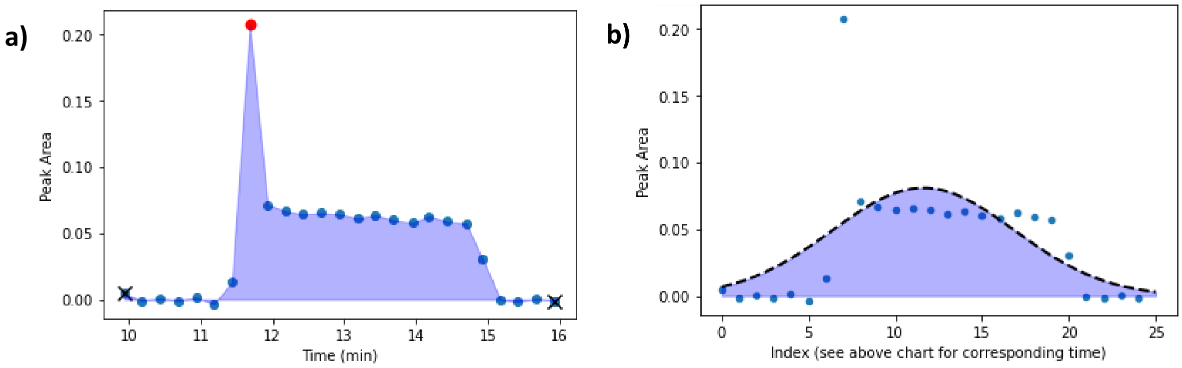

Figure S12: Plots showing the same experimental peak with different peak property picking: a) the red point shows the picked prominence, the blue shaded area shows the area that is integrated; b) the black dotted line shows the best fit gaussian curve with the blue shaded area showing the area that is integrated.

Each property was calculated for each IR peak, the data can be normalized and compared (Figure S13).

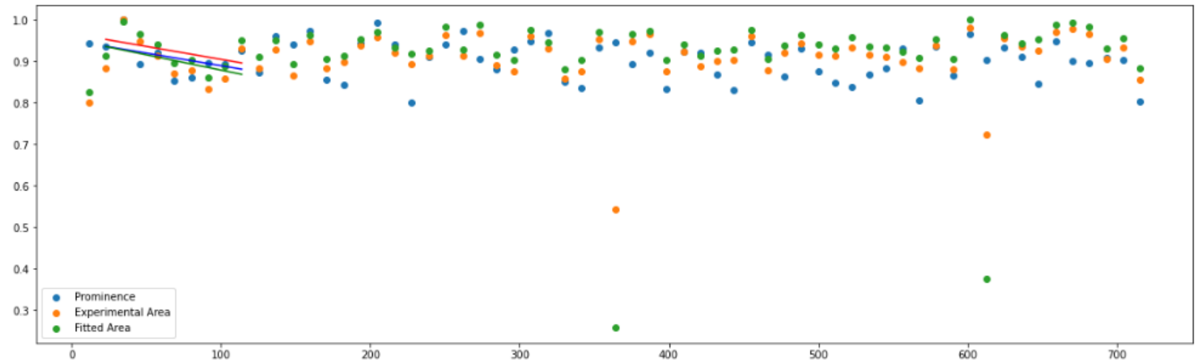

Figure S13: Comparison of normalised peak property data for prominence, experimental area, and fitted area methods.

Box and violin plot analysis shows the distribution of each method (Figure S14).

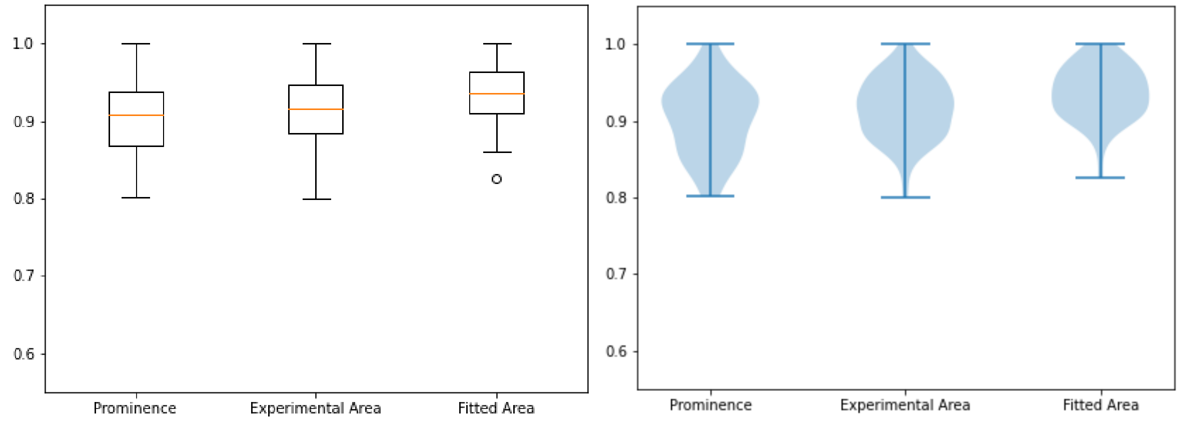

Figure S14: Box and violin plot analysis of normalised data for prominence, experimental area, and fitted area peak property methods.

The median of all methods is relatively similar, giving confidence each method should deliver roughly similar results. Fitted area appears to give the smallest spread, barring an outlier. In practice, the gaussian fitting function required for fitted area integration fails if the initial guesses for fitting were not good enough and was ultimately unreliable for data analysis.

With the behaviour of Prominence and Experimental Area both nearly identical in this analysis, we chose to use Prominence due to the more direct physical interpretation of the reading (a maximum concentration reached in the IR as opposed to the area of an area).

As concentrations decrease at increasing SPKA conversions, IR intensity will also decrease. It was found, as expected, that lower IR intensities involve a smaller degree of absolute error but a larger degree of relative error. Repetition experiments were also carried out at 80%, 50%, and 20% ideal SPKA conversions.

Experimental area was used as the peak property method and box and violin plots for the raw and normalised data were created (Figure S15). The standard deviation for each ideal SPKA conversion can be easily determined.

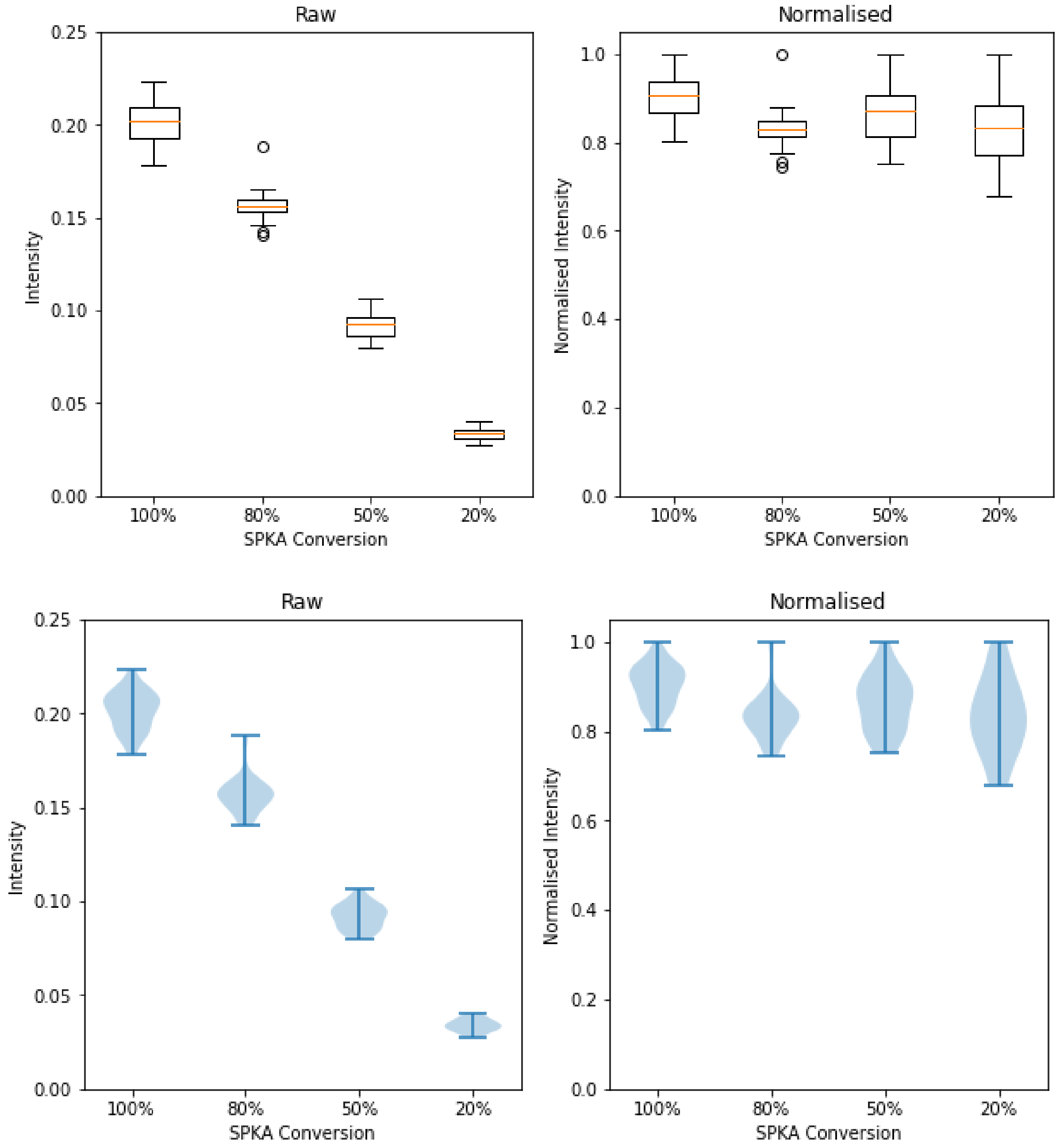

Ideal SPKA Conversion	Standard Deviation (Experimental Area)	Standard Deviation (Prominence)
100%	0.081114	0.010645
80%	0.074629	0.009685
50%	0.037825	0.006698
20%	0.032234	0.003458

Figure S15: Box and violin plots of intensity (left) and normalised intensity (right) of repeated measurements of reaction plugs at different ideal SPKA conversions, including calculated standard deviations.

3.2 Linear Correction

A plot of concentration vs conversion for a reaction should always result in a straight line that must intercept the x-axis when the reaction is complete. This facts allow correction of the experimental error of our platform.

A best fit line can be plotted through the raw data in this manner (Figure S16) and this corrected data can be taken for SPKA analysis. In addition, weighted linear regression, using the experimentally determined standard deviations of Figure S15, allows experimental error at each individual point to be taken into account. Although the differences are small, SPKA, as a differential method, amplifies experimental noise. Linear correction in such a manner is best performed prior to SPKA analysis as this has a rational basis.

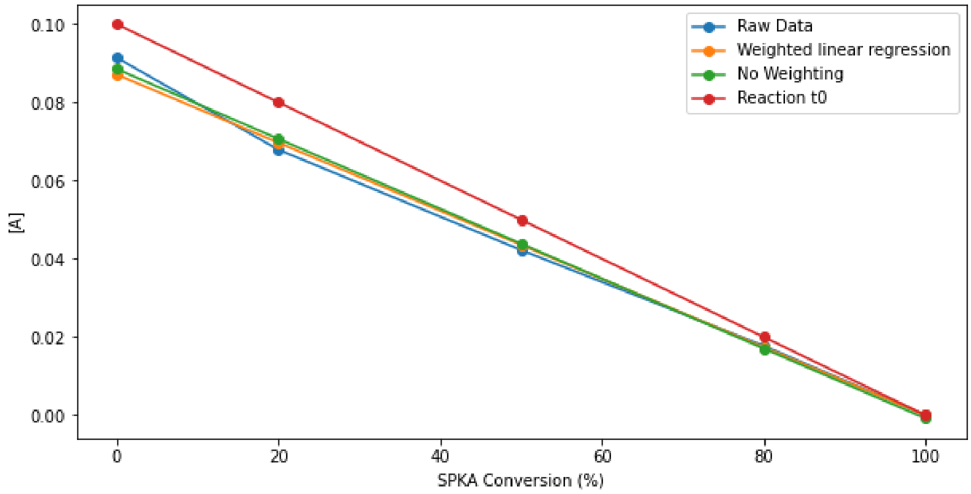

Figure S16: Plot showing raw data, weighted and non-weighted linear corrections, and t0 starting points.

4 Platform Validation

4.1 SPKA Flow vs Batch Data

Blackmond and Armstrong found 0.4M [H_2O] in DMSO necessary to halt the deactivation of proline (**1a**), while also using high loadings (2M) of acetone (**3a**) to combat the inhibiting effects of water.[4] In our platform, high loadings of carbonyl donor led to plug breakdown and beading in to the carrier fluid. To achieve plug stability, the carbonyl donor concentration was reduced. However, this drastically reduced the reaction rate and to combat this the concentration of water was also reduced, making catalyst deactivation, for Proline (**1a**) at least, slowed but not entirely removed. Conditions used for the batch and flow reaction are outlined in Table S4.

Table S4: Batch vs flow conditions.

Reaction	[Aldehyde]	[Ketone]	[Proline]	[H_2O]	[MeOH]
Batch – Standard	0.07	0.15	0.025	0.08	0.56
Batch – Same Excess	0.03	0.11	0.025	0.08	0.56
Flow – SPKA	0.07	0.15	0.025	0.08	0.56
Blackmond and Armstrong[4]	0.5	2	0.014	0.4	0

Due to catalyst deactivation, the reactions in batch take upwards of 16h (Figure S17). A "time-adjusted" same excess protocol is used in reaction progress kinetic analysis (RPKA) to investigate the stability of a catalytic cycle.[5] "Time-adjusted" refers to the arbitrary movement of the second same excess experiment along the x-axis to check for overlay (not observed here, Figure S17). By following this protocol, integral kinetic data can be used rather than differential kinetic data, as is required in the original RPKA method.

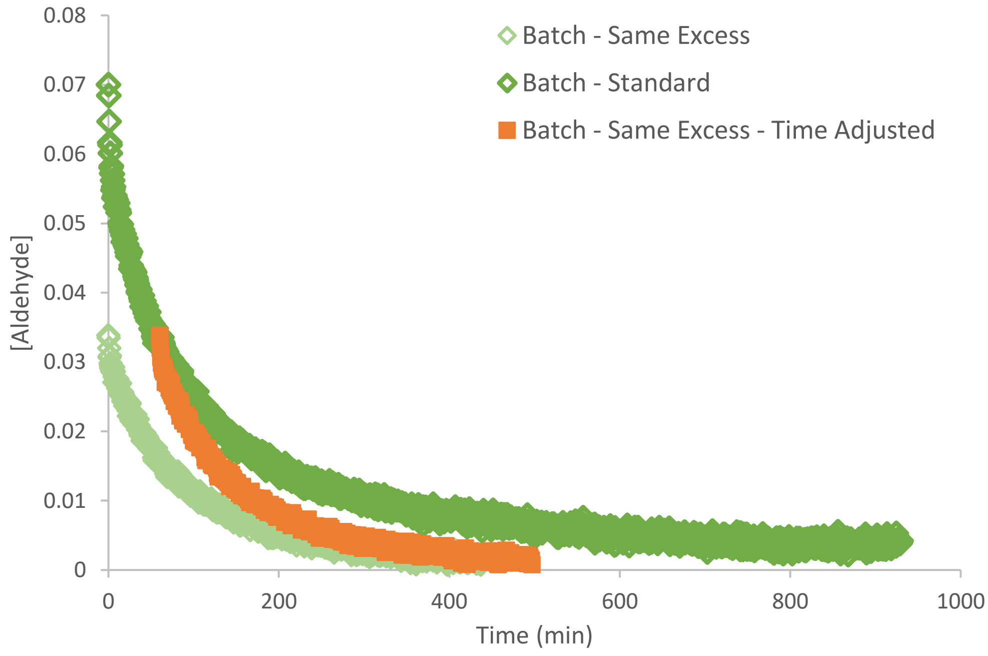

Figure S17: Batch reaction as concentration vs time.

The batch datapoints used to create the initial rates, and so batch SPKA data, are highlighted in red, these are the datapoints 11.4 minutes after reaction initiation, the same as the residence time used in flow (Figure S18).

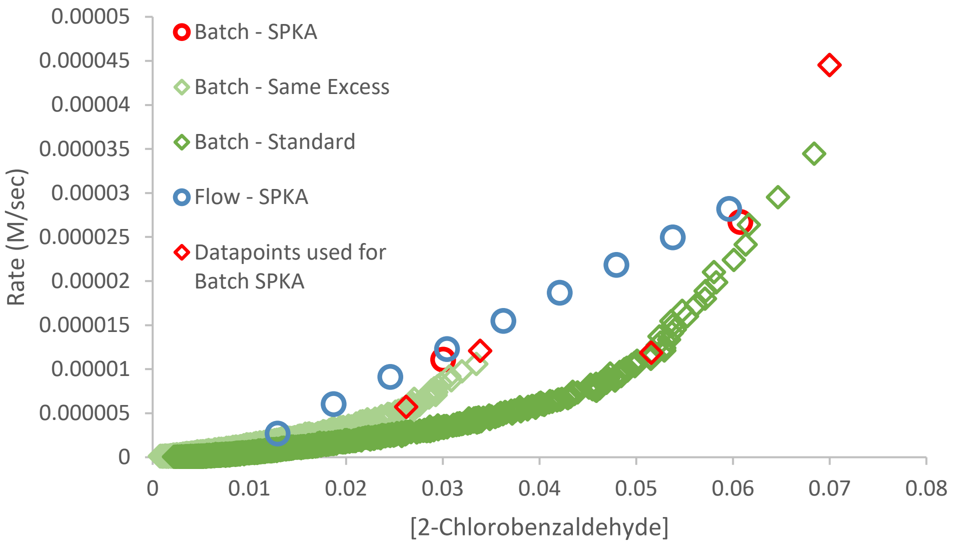

Figure S18: Batch same excess data for the proline mediated aldol reaction showing no overlay meaning catalyst deactivation. Comparison of SPKA rate profiles created in flow and using the batch data, also showing the datapoints used for calculating batch SPKA data.

4.2 Residence Time: Flow Rate vs Coil Length Studies

As outlined in the main body, comparing SPKA profiles collected at different residence times should allow for the interrogation of catalyst stability if there is a lack of overlay (Figure S19a). If a change in residence time is brought about by a change in flowrate, then it is possible that the mixing in flow may also change, influencing the reaction. However, under different flowrates mixing (i.e., Reynolds number) will be different and it is possible that this, or difference in homogeneity of the reaction plugs, could be causing the difference in rate. We collected an SPKA profile using a reactor coil of half the length at flowrates to obtain the same 5- and 10-minute residence times. SPKA profiles collected at different flowrates in the same long and short coils show distinctly different rates (Figure S19a and b). Overlay, or close to, is observed between SPKA profiles at the same residence time but different flowrates in each length of coil (Figure S19c and d), suggesting that mixing and the flow regime have little effect on the SPKA profiles. Each profile was collected three times, the average profile is shown with error bars. Standard deviation in the data is small throughout, showing the repeatability of our platform.

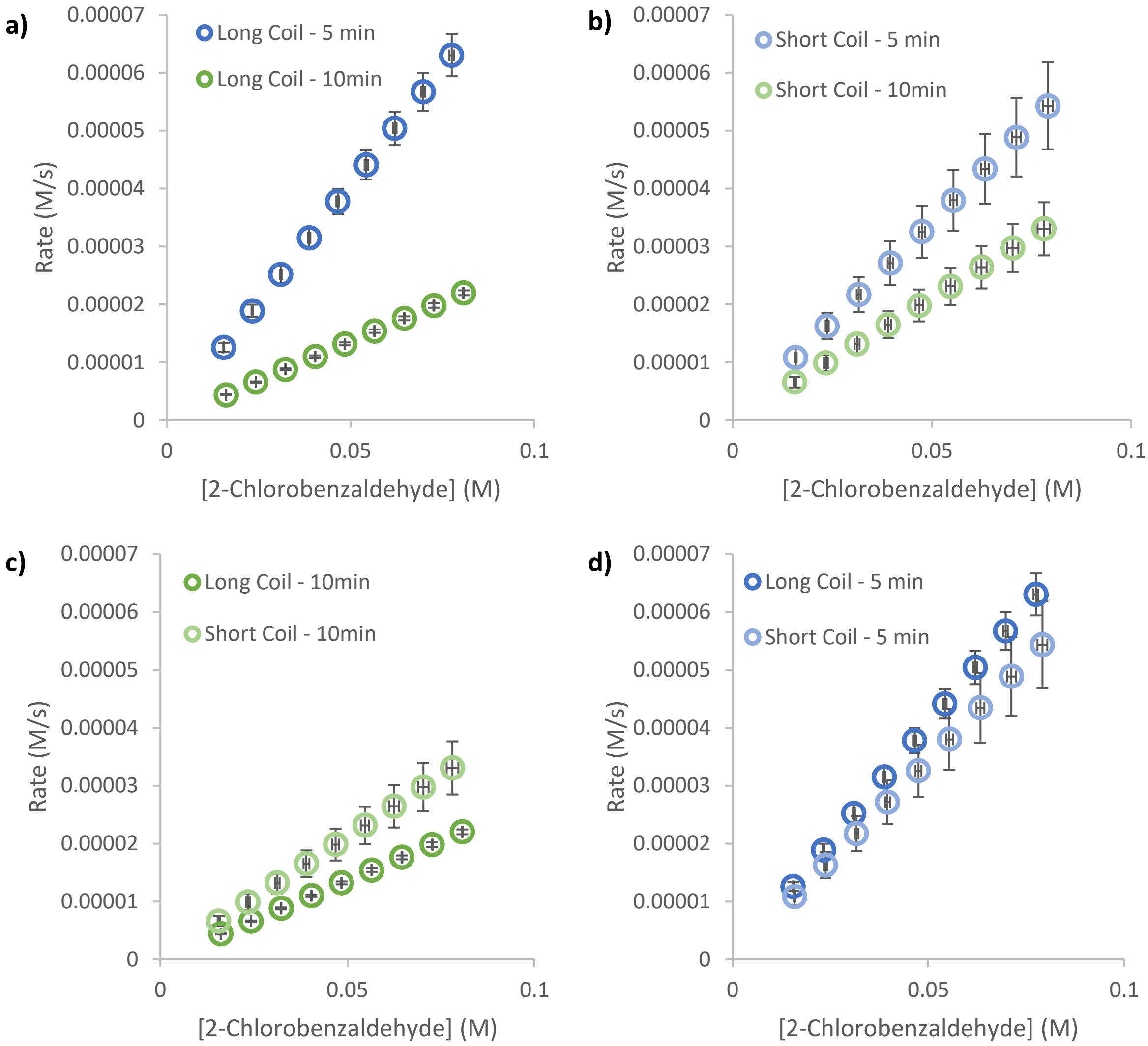

Reactor Coil	Length (m)	5 min Flowrate (mL min^{-1})	10 min Flowrate (mL min^{-1})
Long	3.135	0.554	0.277
Short	1.515	0.277	0.139

Figure S19: Investigating the influence of reactor coil length and flowrate on the SPKA profiles.

4.3 Non-Segmented Flow

We employed segmented flow to keep the concentration of each reaction plug constant throughout its time in the reactor coil. Without segmentation, the reaction plug will axially mix and diffuse into the carrier solvent resulting in a decrease in the reaction concentrations over time. As shown by Sach and co-workers,[3] it is possible to gather single timepoint yield data from reactions in flow in this manner without segmentation. In this example, diffusion into the carrier solvent was used to their advantage, allowing the researchers to investigate the solvent as a reaction parameter without necessitating a change of stock solution solvent.

A platform setup without segmented flow is significantly less complicated (Figure S20) but, clearly, decreasing concentrations in the reaction plug will affect the kinetics. We chose to gather data on the standard Proline-2-Chlorobenzaldehyde-Acetone system and compare the results with and without segmentation.

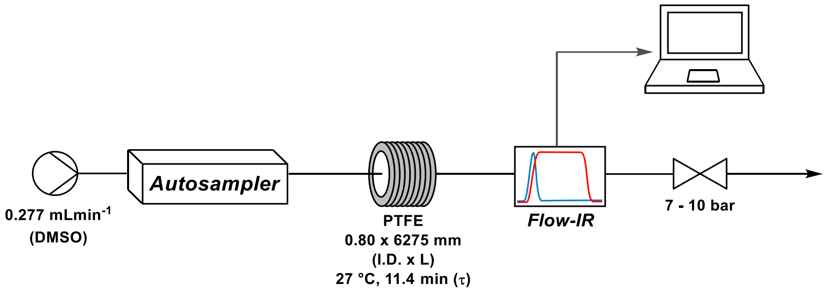

Figure S20: Platform schematic setup without segmentation

A change to reaction preparation was required without segmentation. Under segmented flow, water was introduced to the reaction as part of the carrier solvent. Doing so without segmented flow resulted in very poor-quality data with next to no reactivity observed. We reasoned that this was due to water concentrations staying constant, and high, while all other reaction concentrations decreased. Water has a large negative order on the proline mediated aldol reaction,[6] meaning the reaction was slowing to a stop. Water was, instead, introduced from an additional stock solution during reaction preparation. Besides this, an identical reaction setup (stock solution concentrations and volumes) was used. As a result, absolute reaction rates were found to be, unsurprisingly, lower in non-segmented flow (Figure S21).

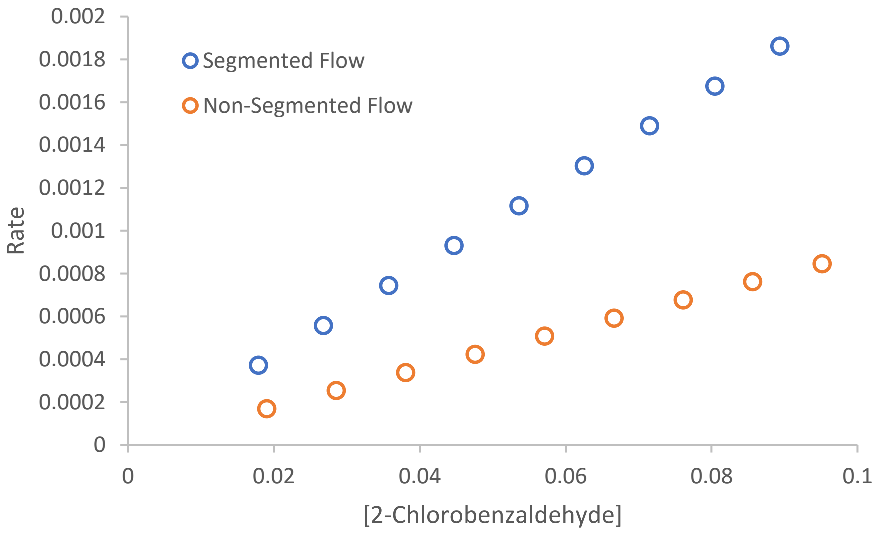

Figure S21: Comparison of kinetic profiles collected under segmented and non-segmented flow.

Despite this, reagent orders, determined by RPKA, were found to be strikingly close between segmented and non-segmented setups (Figure S22).

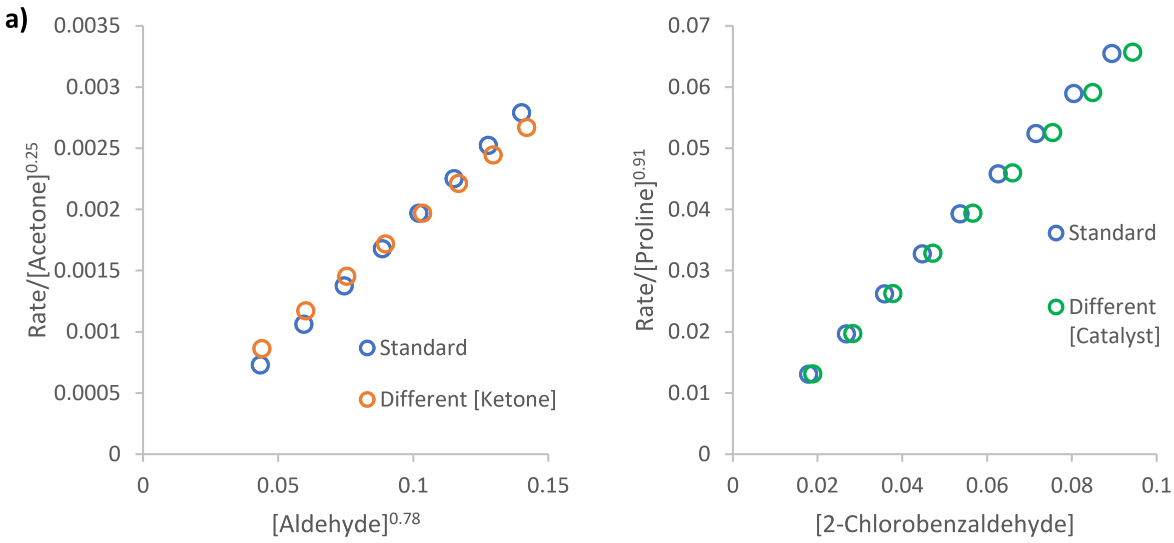

r = k_{obs}[2-Chlorobenzaldehyde]$^{0.78}$[Acetone]$^{0.25}$[Proline]$^{0.91}$

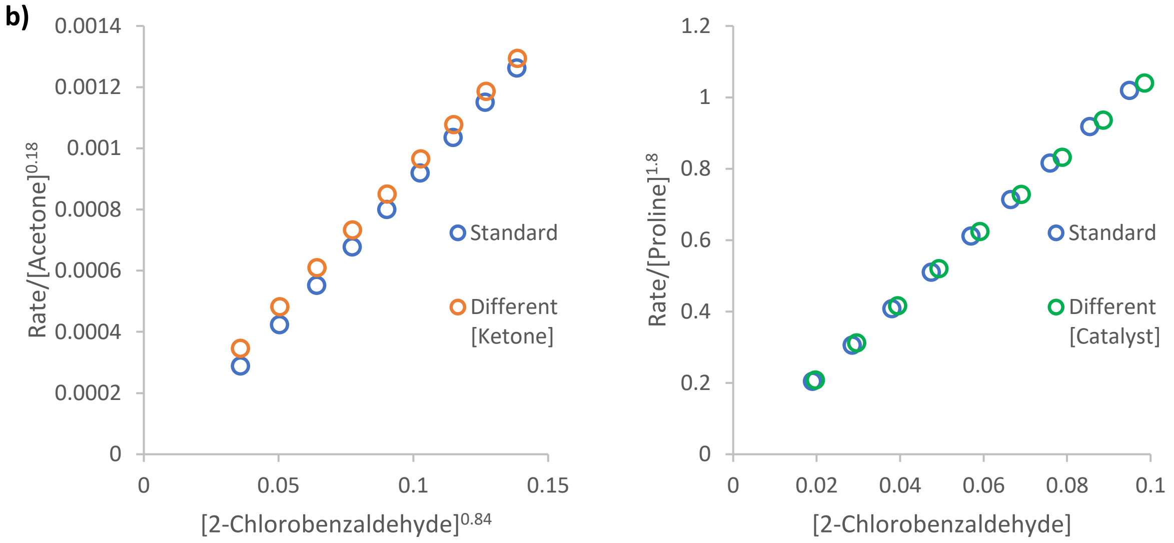

r = k_{obs}[2-Chlorobenzaldehyde]$^{0.84}$[Acetone]$^{0.18}$[Proline]$^{1.8}$

Figure S22: Comparison of reaction orders and rate equations determined by: a) segmented flow; b) non-segmented flow.

5 High Throughput Kinetic Experimentation

5.1 Conditions

Conditions used in the high throughput experimental screen can be found in Table S5. The reaction solvent was DMSO (0.08 M aq.), and the carrier solvent was perfluorodecalin. Reactions were conducted at 29 ˚C. Reaction plug volumes were 150 µl. The total of 216 experiments consumed a total of roughly 162 mL solvent, 1.8 mL of carbonyl acceptor, 1.6 mL of carbonyl donor, and 310 mg of catalyst.

Table S5: Conditions used in high throughput screen.

Reaction	[Aldehyde]	[Ketone]	[Catalyst]	[H_2O]	[MeOH]
1) Standard	0.1	0.15	0.02	0.08	0.56
2) Different [Ketone]	0.1	0.1	0.02	0.08	0.56
3) Different [Catalyst]	0.1	0.15	0.01	0.08	0.56

5.2 Catalyst Stock Solutions

Proline (**1a**) and Proline-OTBS (**1b**) are largely insoluble in DMSO. Instead, methanol was used to create stock solutions of the required concentration (0.9 M). While Proline Tetrazole (**1c**) is reportedly soluble in a much wider range of solvents than Proline (**1a**), it is less soluble in methanol (a maximum of ca. 0.14 M in our hands). However, it is more soluble in DMSO (aq.), up to a maximum of ca. 0.23 M. To utilise proline tetrazole (**1c**) in our studies, the stock solution solvent was switched to DMSA (aq.), and the volume of the catalyst solution used in creating the reaction plugs was increased while the blank DMSO (aq.) solvent was decreased to keep the reaction plugs at the same volumes.

5.3 10 vs 5-point data

Prior to our kinetic and mechanistic screening, we further optimised our platform. Reducing the number of datapoints per profile from ten to five lead to no change in data quality (Figure S23).

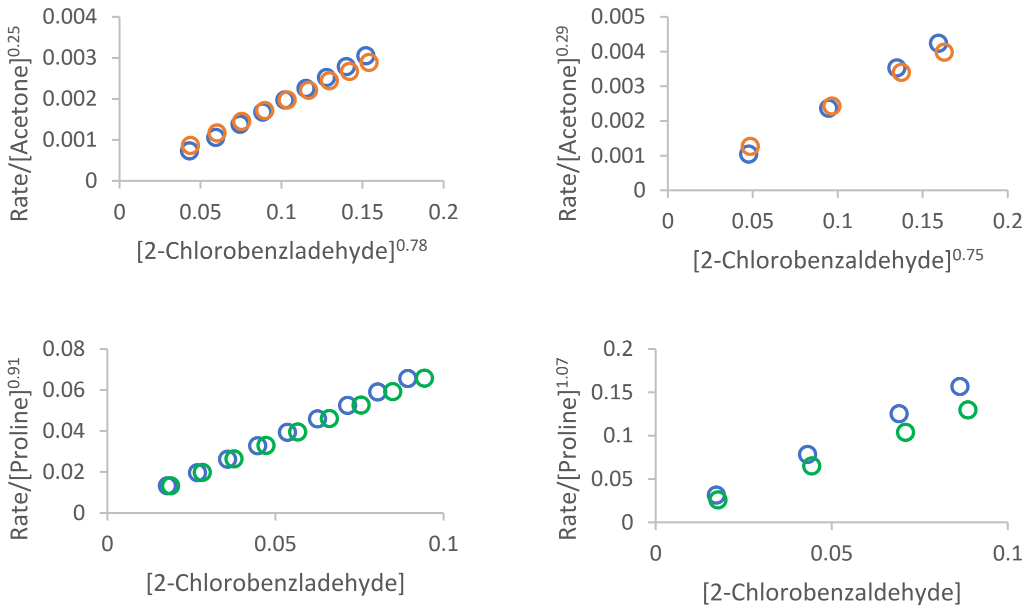

c)

r = k_{obs}[2-Chlorobenzaldehyde]$^{0.9}$[Acetone]$^{0.58}$[Proline]^{1}

Blackmond and Armstrong[4]

r = k_{obs}[2-Chlorobenzaldehyde]$^{0.78}$[Acetone]$^{0.25}$[Proline]$^{0.91}$

SPKA 10-point data

r = k_{obs}[2- Chlorobenzaldehyde]$^{0.75}$[Acetone]$^{0.29}$[Proline]$^{1.07}$

SPKA 5-point data

Figure S23: Determining reagent and catalyst orders using a) 9 data-points; b) 4 data-points; c) a comparison of the determined rate laws.[4] Red: standard reaction; Blue: different [Acetone]; Green: different [Catalyst]. A 10-minute SPKA residence times was used throughout.

5.4 Repeatability

Experiments were run in triplicate, allowing us to investigate the repeatability of the data collected by our platform. Figure S24 shows, as an example, the standard deviations from the HTKE screen of our standard model reaction proline (**1a**), 2-chlorobenzaldehyde (**2a**), acetone (**3a**).

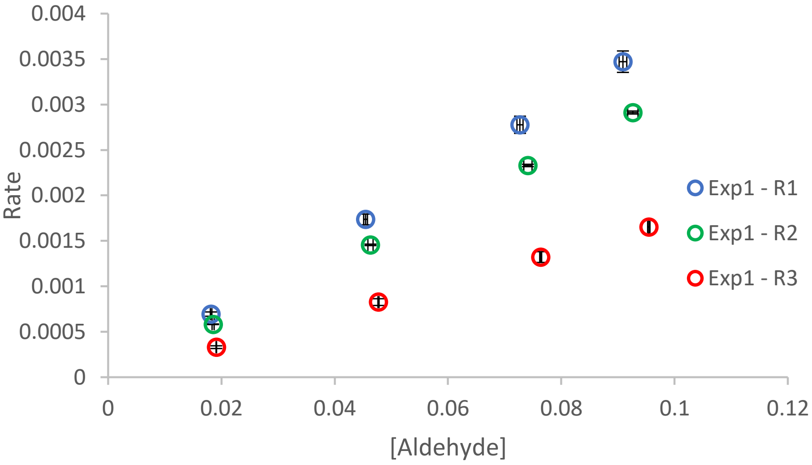

Figure S24: Plot containing standard deviations of the HTKE screen of proline (1a), 2-chlorobenzaldehyde (2a), acetone (3a).

5.5 Practical Limitations and Unsuitable Systems

There are several practical limitations of our current platform. While inline IR is simple to implement, without the need for time consuming method development or separations, the relatively large volumes of perfluorodecalin present means that analyte signals are significantly weaker than in non-segmented flow. This limited us to observing strongly IR active functional groups in the starting material only. As with all flow systems, the platform requires fully soluble reagents. Further, the requirement for stock solutions limits both the maximum concentrations of the reaction as well as the total volume of the reaction plugs.

With these limitations in mind, we found that some systems were unsuitable for use with our platform. While benzaldehyde would have been a useful, and obvious, substrate, we found its IR signal to lay under that of acetone, meaning we were unable to track its reaction. It was not possible to use isovaleraldehyde as, although the reaction did proceed, the IR signal was significantly weaker than aromatic aldehydes used and quickly disappeared into baseline noise. Diphenylprolinol silyl ether, as an important proline derivative, was found to be partially miscible in the perfluorodecalin carrier phase, causing plug breakdown and so could not be used.

5.6 Proline-OTBS (1b) and Proline Tetrazole (1c) Different Residence Time Study

No overlay was observed in a different residence time study for either proline-OTBS (**1b**) or proline tetrazole (**1c**),

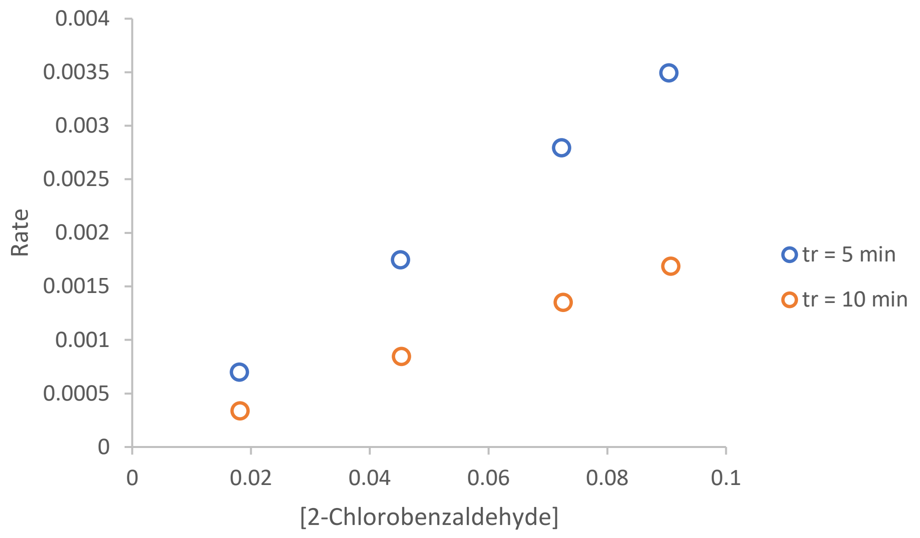

Figure S25: Proline-OTBS (**1b**) different residence time study.

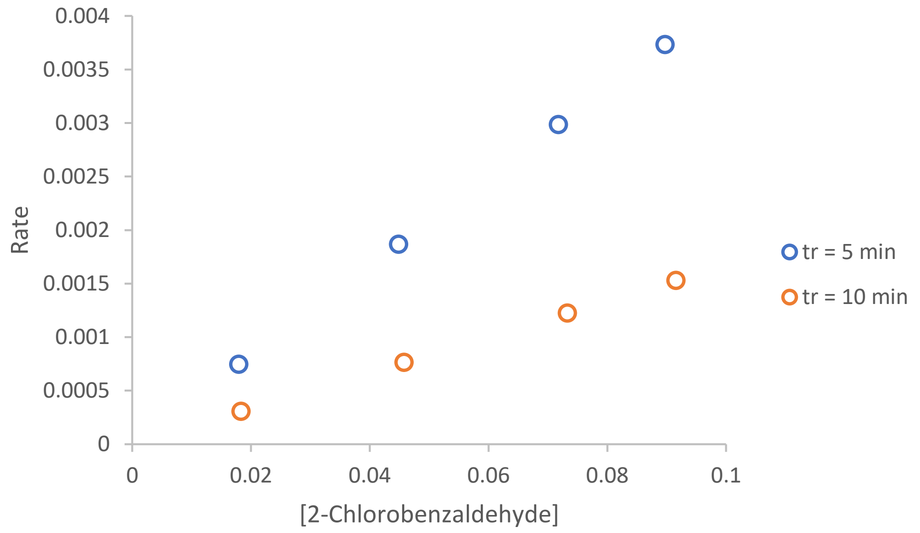

Figure S26: Proline tetrazole (**1c**) different residence time study.

The mechanistic rationale behind each lack of stability of the catalytic cycle for each catalyst differs (Scheme S1), with the double addition product, and overconsumption of the carbonyl acceptor starting material, determined in previous work by Zotova and Blackmond.[7]

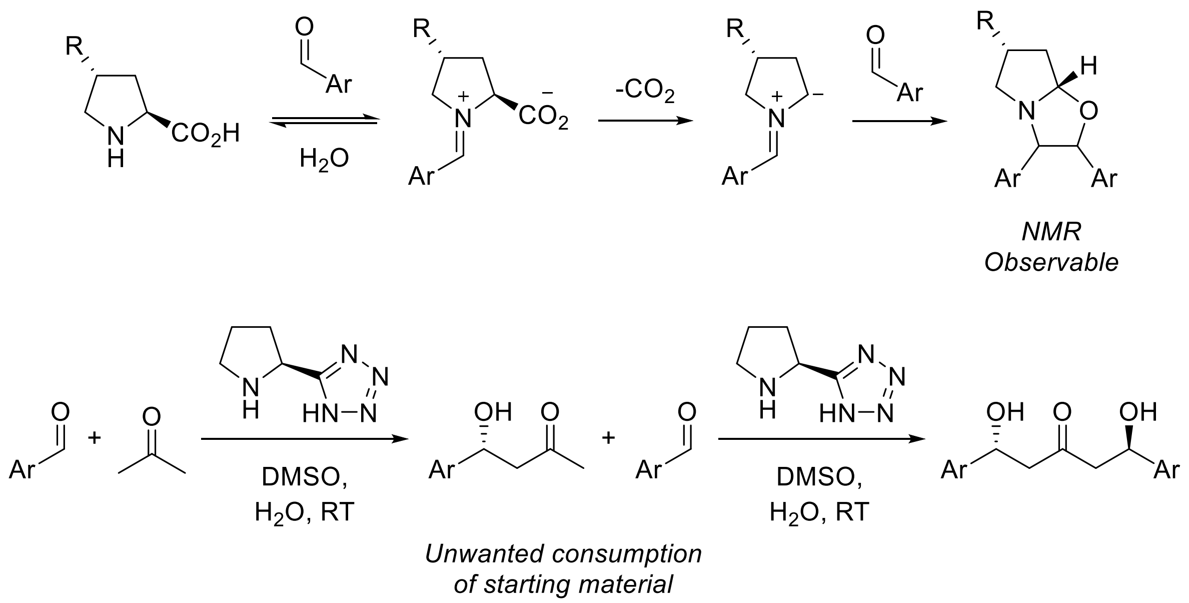

Scheme S1: Mechanistic rationale behind lack of overlay in different residence time plots for different catalysts.

5.7 Data Analysis

5.7.1 3D Plots

Graphical representation of the Table 1 and 2 in the main paper are found in Figure S24 and S25.

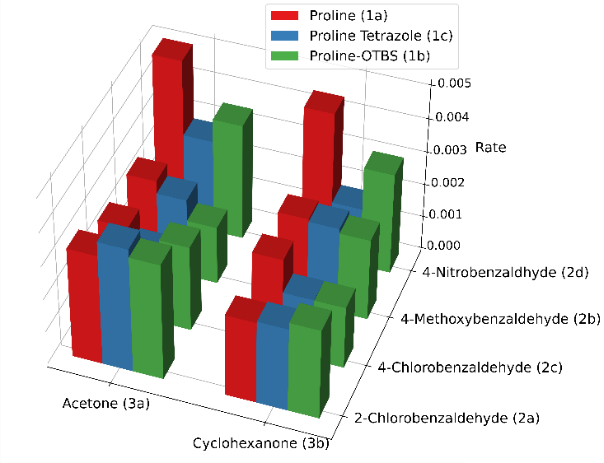

Figure S27: 3D plot of maximum reaction rates from kinetic screen.

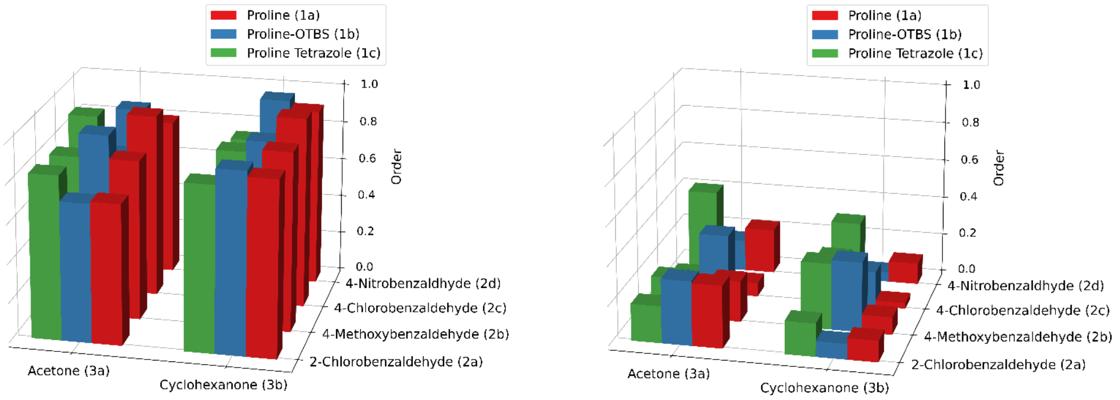

Figure S28: 3D plots of orders for carbonyl acceptor and donor.

The following are pivot tables of different layout to that in the main text. Also included are heatmaps of differing relative rates to attempt to highlight any observed trends.

5.7.2 Carbonyl Donor (Rate)

		Rate (x10^{-3})	
		**Acetone (3a)**	**Cyclohexanone (3b)**
	**Proline (1a)**	3.31	2.46
**2-Cl (2a)**	**Proline Tetrazole (1c)**	3.49	2.70
	**Proline-OTBS (1b)**	3.73	2.51
	**Proline (1a)**	2.74	2.66
**4-OMe (2b)**	**Proline Tetrazole (1c)**	1.74	2.45
	**Proline-OTBS (1b)**	2.37	2.59
	**Proline (1a)**	2.73	2.86
**4-Cl (3c)**	**Proline Tetrazole (1c)**	2.56	1.87
	**Proline-OTBS (1b)**	2.15	1.87
**4-NO2 (3d)**	**Proline (1a)**	5.04	4.46

	**Proline Tetrazole (1c)**	3.50	3.04
	**Proline-OTBS (1b)**	2.81	1.78

		**Relative Rate**	
		**Acetone (3a)**	**Cyclohexanone (3b)**
**2-Cl (2a)**	**Proline (1a)**	1.00	0.74
	**Proline Tetrazole (1c)**	1.00	0.67
	**Proline-OTBS (1b)**	1.00	0.77
**4-OMe (2b)**	**Proline (1a)**	1.00	0.97
	**Proline Tetrazole (1c)**	1.00	1.09
	**Proline-OTBS (1b)**	1.00	1.41
**4-Cl (3c)**	**Proline (1a)**	1.00	1.05
	**Proline Tetrazole (1c)**	1.00	0.87
	**Proline-OTBS (1b)**	1.00	0.73
**4-NO2 (3d)**	**Proline (1a)**	1.00	0.89
	**Proline Tetrazole (1c)**	1.00	0.63
	**Proline-OTBS (1b)**	1.00	0.87

5.7.3 Carbonyl Acceptor (Rate)

		Rate (x10^{-3})			
		2-Cl (2a)	4-OMe (2b)	4-Cl (3c)	4-NO2 (3d)
Acetone (3a)	Proline (1a)	3.31	2.74	2.73	5.04
	Proline-OTBS (1b)	3.49	1.74	2.56	3.50
	Proline Tetrazole (1c)	3.73	2.37	2.15	2.81
Cyclohexanone (3b)	Proline (1a)	2.46	2.66	2.86	4.46
	Proline-OTBS (1b)	2.70	2.45	1.87	3.04
	Proline Tetrazole (1c)	2.51	2.59	1.87	1.78

		Relative Rate			
		2-Cl (2a)	4-OMe (2b)	4-Cl (3c)	4-NO2 (3d)
Acetone (3a)	Proline (1a)	1.00	0.83	0.82	1.52
	Proline-OTBS (1b)	1.00	0.50	0.73	1.00
	Proline Tetrazole (1c)	1.00	0.64	0.58	0.75
Cyclohexanone (3b)	Proline (1a)	1.00	1.08	1.16	1.81
	Proline-OTBS (1b)	1.00	0.91	0.69	1.12
	Proline Tetrazole (1c)	1.00	1.03	0.75	0.71

		Relative Rate			
		2-Cl (2a)	4-OMe (2b)	4-Cl (3c)	4-NO2 (3d)
Acetone (3a)	Proline (1a)	1.21	1.00	1.00	1.84
	Proline-OTBS (1b)	2.01	1.00	1.47	2.01
	Proline Tetrazole (1c)	1.57	1.00	0.91	1.19
Cyclohexanone (3b)	Proline (1a)	0.93	1.00	1.08	1.67
	Proline-OTBS (1b)	1.10	1.00	0.76	1.24
	Proline Tetrazole (1c)	0.97	1.00	0.72	0.69

		Relative Rate			
		2-Cl (2a)	4-OMe (2b)	4-Cl (3c)	4-NO2 (3d)
Acetone (3a)	Proline (1a)	0.66	0.54	0.54	1.00
	Proline-OTBS (1b)	1.00	0.50	0.73	1.00
	Proline Tetrazole (1c)	1.33	0.84	0.76	1.00
Cyclohexanone (3b)	Proline (1a)	0.55	0.60	0.64	1.00
	Proline-OTBS (1b)	0.89	0.81	0.61	1.00
	Proline Tetrazole (1c)	1.41	1.45	1.05	1.00

5.7.4 Catalyst (Rate)

		Relative Rate		
		Proline (1a)	Proline-OTBS (1b)	Proline Tetrazole (1c)
Acetone (3a)	2-Cl (2a)	1.00	1.05	1.13
	4-OMe (2b)	1.00	0.63	0.86
	4-Cl (3c)	1.00	0.94	0.79
	4-NO2 (3d)	1.00	0.69	0.56
Cyclohexanone (3b)	2-Cl (2a)	1.00	1.10	1.02
	4-OMe (2b)	1.00	0.92	0.97
	4-Cl (3c)	1.00	0.65	0.65
	4-NO2 (3d)	1.00	0.68	0.40

		Relative Rate		
		Proline (1a)	Proline-OTBS (1b)	Proline Tetrazole (1c)
Acetone (3a)	2-Cl (2a)	0.95	1.00	1.07
	4-OMe (2b)	1.58	1.00	1.36
	4-Cl (3c)	1.07	1.00	0.84
	4-NO2 (3d)	1.44	1.00	0.80
Cyclohexanone (3b)	2-Cl (2a)	0.91	1.00	0.93
	4-OMe (2b)	1.09	1.00	1.06
	4-Cl (3c)	1.53	1.00	1.00
	4-NO2 (3d)	1.47	1.00	0.59

		Relative Rate		
		Proline (1a)	Proline-OTBS (1b)	Proline Tetrazole (1c)
Acetone (3a)	2-Cl (2a)	0.89	0.94	1.00
	4-OMe (2b)	1.16	0.73	1.00
	4-Cl (3c)	1.27	1.19	1.00
	4-NO2 (3d)	1.79	1.24	1.00
Cyclohexanone (3b)	2-Cl (2a)	0.98	1.08	1.00
	4-OMe (2b)	1.03	0.95	1.00
	4-Cl (3c)	1.53	1.00	1.00
	4-NO2 (3d)	2.51	1.71	1.00

5.8 Reagent Order Analysis

		Order in Catalyst		
		Proline (1a)	Proline-OTBS (1b)	Proline Tetrazole (1c)
Acetone (3a)	2-Cl (2a)	1.00	0.77	0.51
	4-OMe (2b)	0.51	0.52	0.50
	4-Cl (3c)	0.50	1.00	0.84
	4-NO2 (3d)	0.57	0.51	0.56
Cyclohexanone (3b)	2-Cl (2a)	0.51	0.52	0.51
	4-OMe (2b)	0.51	0.51	0.52
	4-Cl (3c)	0.51	0.51	0.50
	4-NO2 (3d)	0.50	0.51	0.50

		Order in A			Order in B		
		Proline (1a)	Proline-OTBS (1b)	Proline Tetrazole (1c)	Proline (1a)	Proline-OTBS (1b)	Proline Tetrazole (1c)
Acetone (3a)	2-Cl (2a)	1.00	0.99	1.17	1.00	1.03	0.59
	4-OMe (2b)	1.00	1.15	1.00	1.00	0.29	0.95
	4-Cl (3c)	1.00	0.78	0.98	1.00	4.43	1.29
	4-NO2 (3d)	1.00	1.08	0.80	1.00	0.70	1.78
Cyclohexanone (3b)	2-Cl (2a)	1.00	1.03	0.94	1.00	0.73	1.55
	4-OMe (2b)	1.00	0.74	0.76	1.00	4.00	3.78
	4-Cl (3c)	1.00	0.87	0.81	1.00	6.00	8.00
	4-NO2 (3d)	1.00	1.05	0.81	1.00	0.45	2.73

		Order in A			Order in B		
		Proline (1a)	Proline-OTBS (1b)	Proline Tetrazole (1c)	Proline (1a)	Proline-OTBS (1b)	Proline Tetrazole (1c)
Acetone (3a)	2-Cl (2a)	1.01	1.00	1.18	0.97	1.00	0.58
	4-OMe (2b)	0.87	1.00	0.87	3.50	1.00	3.33
	4-Cl (3c)	1.29	1.00	1.26	0.23	1.00	0.29
	4-NO2 (3d)	0.93	1.00	0.74	1.44	1.00	2.56
Cyclohexanone (3b)	2-Cl (2a)	0.97	1.00	0.91	1.38	1.00	2.13
	4-OMe (2b)	1.35	1.00	1.03	0.25	1.00	0.94
	4-Cl (3c)	1.15	1.00	0.93	0.17	1.00	1.33
	4-NO2 (3d)	0.95	1.00	0.77	2.20	1.00	6.00

		Order in A			Order in B		
		Proline (1a)	Proline-OTBS (1b)	Proline Tetrazole (1c)	Proline (1a)	Proline-OTBS (1b)	Proline Tetrazole (1c)
Acetone (3a)	2-Cl (2a)	0.86	0.85	1.00	1.68	1.74	1.00
	4-OMe (2b)	1.00	1.15	1.00	1.05	0.30	1.00
	4-Cl (3c)	1.02	0.79	1.00	0.78	3.44	1.00
	4-NO2 (3d)	1.25	1.34	1.00	0.56	0.39	1.00
Cyclohexanone (3b)	2-Cl (2a)	1.06	1.09	1.00	0.65	0.47	1.00
	4-OMe (2b)	1.31	0.97	1.00	0.26	1.06	1.00
	4-Cl (3c)	1.24	1.08	1.00	0.13	0.75	1.00
	4-NO2 (3d)	1.23	1.30	1.00	0.37	0.17	1.00

6 References

[1] S. Hoops, S. Sahle, R. Gauges, C. Lee, J. Pahle, N. Simus, M. Singhal, L. Xu, P. Mendes, U. Kummer, *Bioinformatics* **2006**, *22*, 3067-3074.

[2] N. Hawbaker, E. Wittgrove, B. Christensen, N. Sach, D. G. Blackmond, *Org. Process Res. Dev.* **2016**, *20*, 465-473.

[3] D. Perera, J. W. Tucker, S. Brahmbhatt, C. J. Helal, A. Chong, W. Farrell, P. Richardson, N. W. Sach, *Science* **2018**, *359*, 429-434.

[4] N. Zotova, L. J. Broadbelt, A. Armstrong, D. G. Blackmond, *Bioorganic Med. Chem. Lett.* **2009**, *19*, 3934-3937.

[5] (a) D. G. Blackmond, *Angew. Chem. Int. Ed.* **2005**, *44*, 4302-4320; (b) R. D. Baxter, D. Sale, K. M. Engle, J. Q. Yu, D. G. Blackmond, *J. Am. Chem. Soc.* **2012**, *134*, 4600-4606.

[6] N. Zotova, A. Franzke, A. Armstrong, D. G. Blackmond, *J. Am. Chem. Soc.* **2007**, *129*, 15100-15101.

[7] N. Zotova, Thesis (Ph.D.) thesis, Imperial College London (Imperial College London), **2008**.
